# Author Correction: Aplp1 interacts with Lag3 to facilitate transmission of pathologic α-synuclein

**DOI:** 10.1038/s41467-024-50640-2

**Published:** 2024-07-30

**Authors:** Xiaobo Mao, Hao Gu, Donghoon Kim, Yasuyoshi Kimura, Ning Wang, Enquan Xu, Ramhari Kumbhar, Xiaotian Ming, Haibo Wang, Chan Chen, Shengnan Zhang, Chunyu Jia, Yuqing Liu, Hetao Bian, Senthilkumar S. Karuppagounder, Fatih Akkentli, Qi Chen, Longgang Jia, Heehong Hwang, Su Hyun Lee, Xiyu Ke, Michael Chang, Amanda Li, Jun Yang, Cyrus Rastegar, Manjari Sriparna, Preston Ge, Saurav Brahmachari, Sangjune Kim, Shu Zhang, Yasushi Shimoda, Martina Saar, Haiqing Liu, Sin Ho Kweon, Mingyao Ying, Creg J. Workman, Dario A. A. Vignali, Ulrike C. Muller, Cong Liu, Han Seok Ko, Valina L. Dawson, Ted M. Dawson

**Affiliations:** 1 Neuroregeneration and Stem Cell Programs, Institute for Cell Engineering, Johns Hopkins University School of Medicine, Baltimore, MD 21205 USA; 2 Department of Neurology, Johns Hopkins University School of Medicine, Baltimore, MD 21205 USA; 3 Adrienne Helis Malvin Medical Research Foundation, New Orleans, LA 70130-2685 USA; 4 Interdisciplinary Research Center on Biology and Chemistry, Shanghai Institute of Organic Chemistry, Chinese Academy of Sciences, 26 Qiuyue Road, Shanghai, 201210 China; 5 University of the Chinese Academy of Sciences, 19 A Yuquan Road, Shijingshan District, Beijing, 100049 China; 6 Institute for NanoBioTechnology, Johns Hopkins University, Baltimore, MD 21218 USA; 7 Department of Materials Science and Engineering, Whiting School of Engineering, Johns Hopkins University, Baltimore, MD 21218 USA; 8 Department of Bioengineering, Nagaoka University of Technology, 1603-1 Kamitomiokamachi, Nagaoka, Niigata, 940-2188 Japan; 9 Institute for Pharmacy and Molecular Biotechnology IPMB, Department of Functional Genomics, University of Heidelberg, Im Neuenheimer Feld 364, 69120 Heidelberg, Germany; 10 Hugo W. Moser Research Institute at Kennedy Krieger, 707 North Broadway, Baltimore, MD 21205 USA; 11 Department of Immunology, University of Pittsburgh School of Medicine, Pittsburgh, PA 15261 USA; 12 Tumor Microenvironment Center, UPMC Hillman Cancer Center, Pittsburgh, PA 15232 USA; 13 Department of Physiology, Johns Hopkins University School of Medicine, Baltimore, MD 21205 USA; 14 Solomon H. Snyder Department of Neuroscience, Johns Hopkins University School of Medicine, Baltimore, MD 21205 USA; 15 Department of Pharmacology and Molecular Sciences, Johns Hopkins University School of Medicine, Baltimore, MD 21205 USA; 16 Present Address: Department of Neurology, Nanjing Brain Hospital, Nanjing, Jiangsu 210029 PR China; 17 Present Address: Medical College, Yangzhou University, Yangzhou, Jiangsu 225001 PR China; 18 Present Address: Department of Anesthesiology, West China Hospital, Sichuan University. The Research Units of West China (2018RU012)-Chinese Academy of Medical Sciences, West China Hospital, Sichuan University, Chengdu, Sichuan 610041 PR China; 19 Present Address: Department of Brain and Cognitive Sciences, Massachusetts Institute of Technology, Cambridge, MA 02139 USA; 20 Present Address: Department of Pharmacology, College of Medicine, Dong-A University, 32 Daesin Gongwwon-ro, Seo-gu, Busan, 49201 Republic of Korea; 21 Present Address: Department of Biological Science and Biotechnology, Chungbuk National University, Cheongju, Chungbuk 28644 Republic of Korea; 22 Present Address: Department of Physiology, School of Basic Medical Sciences (Institute of Basic Medical Sciences), Shandong First Medical University & Shandong Academy of Medical Sciences, Jinan, 250000 China; 23 Present Address: Picower Institute for Learning and Memory, Cambridge, MA 02139 USA; 24 Present Address: Harvard-MIT MD/PhD Program, Harvard Medical School, Boston, MA 02115 USA

**Keywords:** Cellular neuroscience, Parkinson's disease, Parkinson's disease, Molecular neuroscience

Correction to: *Nature Communications* 10.1038/s41467-024-49016-3, published online 31 May 2024

The original version of this Article contained an error in Fig. 4, in which the statistical significance for conditions 410C9 versus Lag3^−/−^ in panel 4i was reported as not significant when the association is significant.

The correct version of Fig 4 is:
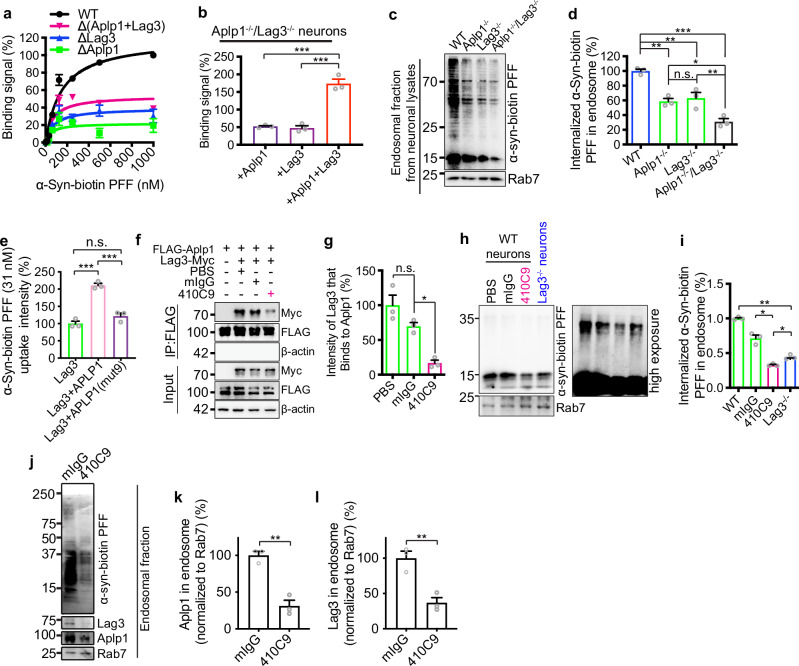


Which replaces the previous incorrect version:
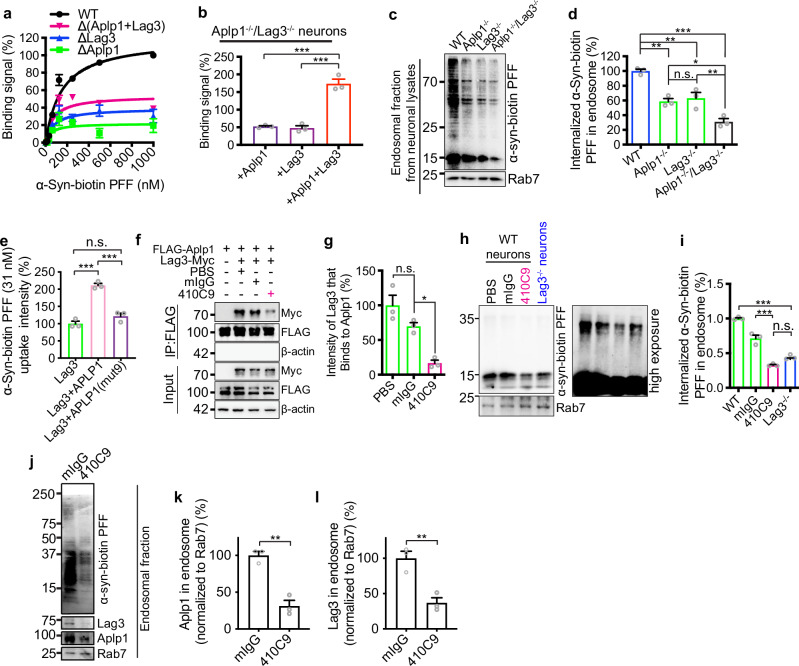


The original version of this Article contained an error in the figure legend of Fig. 4 describing panels h and i, which incorrectly read ‘Anti-Lag3 410C9 (330 nM) reduced the internalization of α-syn-biotin PFF (1 μM) in WT neurons compared to Lag3^–/–^ neurons. Quantification of the intensity of internalized α-syn-biotin PFF normalized by Rab7. (*p*-values: WT vs. *Lag3*^−/−^ < 0.0001, mIgG vs. 410C9 < 0.0001, mIgG vs. *Lag3*^−/−^ 0.0004, 410C9 vs. *Lag3*^−/−^ 0.0641)’. The correct version replaces this with ‘Anti-Lag3 410C9 (330 nM) reduced the internalization of α-syn-biotin PFF (1 μM) in WT neurons compared to Lag3^–/–^ neurons. Quantification of the intensity of internalized α-syn-biotin PFF normalized by Rab7. (p-values: WT vs. Lag3^−/−^ < 0.0057, mIgG vs. 410C9 < 0.0277, 410C9 vs. Lag3^−/−^ 0.0152).’

This has been corrected in both the PDF and HTML versions of the Article.

