# Supplementary material for: Aplp1 interacts with Lag3 to facilitate transmission of pathologic α-synuclein
Source: Nat Commun. 2024 May 31;15:4663. doi: 10.1038/s41467-024-49016-3 (PMC11143359; doi:10.1038/s41467-024-49016-3)

Supplementary Information

for

Aplp1 interacts with Lag3 to facilitates transmission of pathologic α-synuclein

Authors: Xiaobo Mao[1,2,3,†,*], Hao Gu[1,2,†,‡,§], Donghoon Kim[1,2,†,€] Yasuyoshi Kimura[1,2], Ning Wang[1,2] , Enquan Xu[1,2], Ramhari Kumbhar[1,2,3,], Xiaotian Ming[1,2], Haibo Wang[1,2], Chan Chen[1,2,||], Shengnan Zhang[4], Chunyu Jia[4,5], Yuqing Liu[1,2], Hetao Bian[1,2], Senthilkumar S. Karuppagounder[1,2], Fatih Akkentli[1,2,3], Qi Chen[1,2], Longgang Jia[1,2], Heehong Hwang[1,2], Su Hyun Lee[1,2], Xiyu Ke[6,7], Michael Chang[1,2], Amanda Li[1,2], Jun Yang[1,2], Cyrus Rastegar[1,2], Manjari Sriparna[1,2], Preston Ge[1,2,¶] , Saurav Brahmachari[1,2], Sangjune Kim[1,2,#], Shu Zhang[1,2], Yasushi Shimoda[8], Martina Saar[9], Haiqing Liu[1,2ω], Sin Ho Kweon[1,2], Mingyao Ying[2,10], Creg J. Workman[11], Dario A. A. Vignali[11,12], Ulrike C. Muller[9], Cong Liu[4], Han Seok Ko[1,2,3*], Valina L. Dawson[1,2,3,13,14*], Ted M. Dawson[1,2,3,14,15*]

*Corresponding authors: Email: tdawson@jhmi.edu (TMD); vdawson1@jhmi.edu (VLD); hko3@jhmi.edu (HSK); xmao4@jhmi.edu (XM);

The PDF file includes

Supplementary Figures and Figure Legends

Supplementary Fig.1 Aplp1 binds to α-syn-biotin PFF but not monomer.

Supplementary Fig. 2 α-Syn PFF binds to Aplp1 in cell surface binding assay.

Supplementary Fig. 3 Lag3 is expressed in neurons in WT mice.

Supplementary Fig. 4 Lag3 is detected in the neurons by RNAscope.

Supplementary Fig. 5 Lag3 is expressed in neurons in *Lag3*$^{L/L-YFP}$ mice.

Supplementary Fig. 6 α-Syn PFF binding to primary neurons overexpressing Aplp1 and Lag3.

Supplementary Fig. 7 α-Syn PFF binding to Lag3 and Aplp1.

Supplementary Fig. 8 Deletion of Aplp1 and Aplp1-Lag3 prevent neurodegeneration induced by α-syn PFF.

Supplementary Fig. 9 Anti-Lag3 410C9 blocks neurodegeneration induced by α-syn PFF *in vivo*.

Supplementary Table 1: List of Oligonucleotides used in this study

Supplementary Table 2: List of antibodies used in this study

Uncropped blots

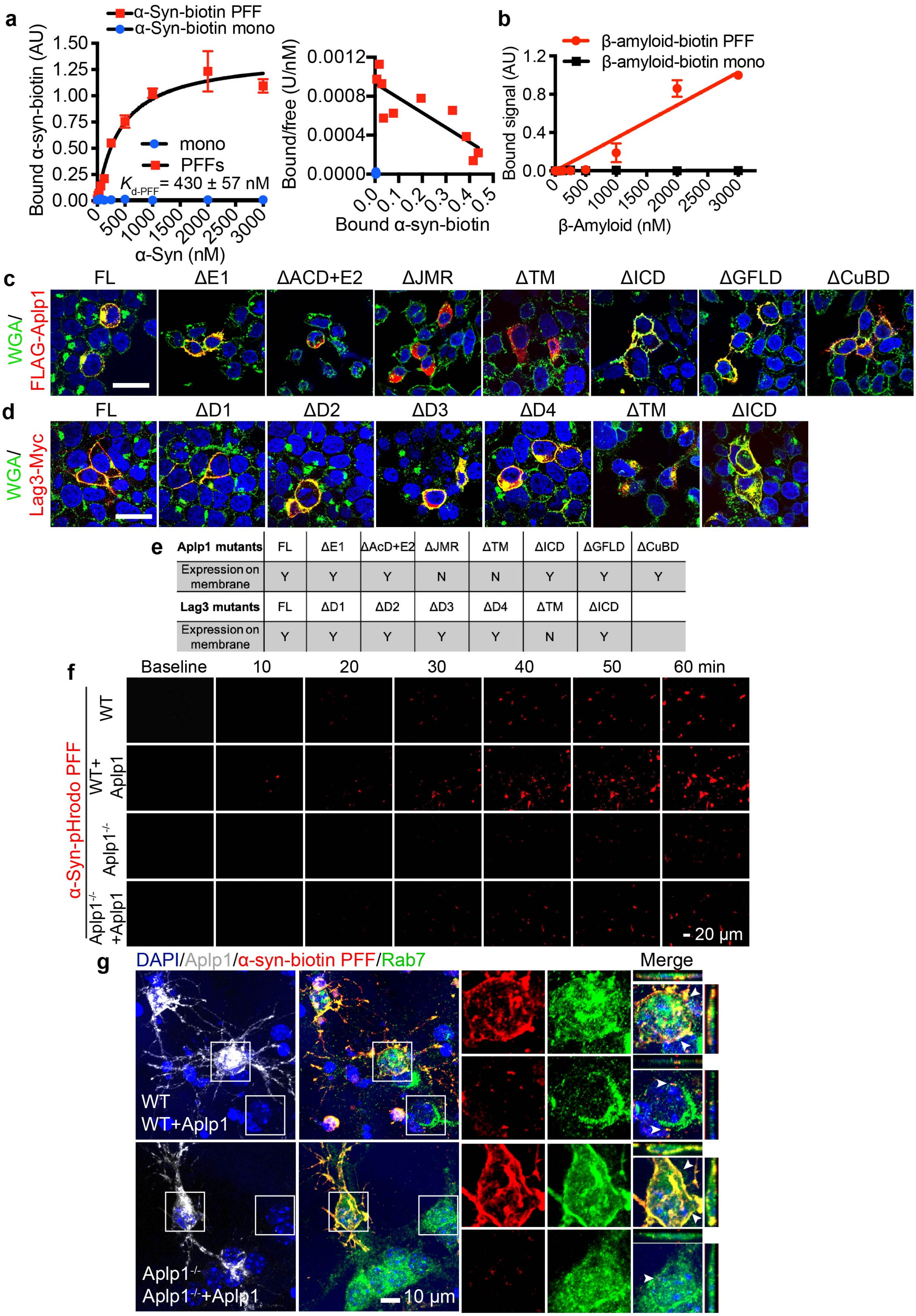
a
α-Syn-biotin PFF
α-Syn-biotin mono
Bound α-syn-biotin (AU)
1.50
1.25
1.00
0.75
0.50
0.25
0.00
mono
PFFs
K d-PFF = 430 ± 57 nM
0
500
1000
1500
2000
2500
3000
α-Syn (nM)
Bound/free (U/nM)
0.0012
0.0008
0.0004
0.0000
0.0
0.1
0.2
0.3
0.4
0.5
Bound α-syn-biotin
b
β-amyloid-biotin PFF
β-amyloid-biotin mono
Bound signal (AU)
1.2
0.8
0.4
0.0
β-Amyloid (nM)
c
WGA/ FLAG-Aplp1
FL
ΔE1
ΔACD+E2
ΔJMR
ΔTM
ΔICD
ΔGFLD
ΔCuBD
d
WGA/ Lag3-Myc
FL
ΔD1
ΔD2
ΔD3
ΔD4
ΔTM
ΔICD
e
Aplp1 mutants | FL | ΔE1 | ΔAcD+E2 | ΔJMR | ΔTM | ΔICD | ΔGFLD | ΔCuBD
Expression on membrane | Y | Y | Y | N | N | Y | Y | Y
Lag3 mutants | FL | ΔD1 | ΔD2 | ΔD3 | ΔD4 | ΔTM | ΔICD
Expression on membrane | Y | Y | Y | Y | Y | N | Y
f
Baseline
10
20
30
40
50
60 min
α-Syn-pHrodo PFF
WT
WT+ Aplp1
Aplp1-/-
Aplp1-/- +Aplp1
20 μm
g
DAPI/Aplp1/α-syn-biotin PFF/Rab7
Merge
WT
WT+Aplp1
Aplp1-/-
Aplp1-/-+Aplp1
10 μm

Supplementary Fig.1 | Aplp1 binds to α-syn-biotin PFF but not monomer. a, α-Syn-biotin PFF binding to SH-SY5Y cells expressing Aplp1 as a function of α-syn concentration with Scatchard analysis. α-Syn-biotin monomer has minimal binding affinity with cells. $K_{d\text{-PFF}}$ = 430 ± 57 nM for PFF, data are the means ± SEM, N = 3 independent experiments. **b**, β-Amyloid PFF binds to Aplp1 in non-specific manner (K_d > 3000 nM), but β-amyloid monomer does not exhibit appreciable binding to Aplp1. **c**,**d**,**e**, The membrane localization of Aplp1 and Lag3 mutants. **c**, The anti-FLAG immunostaining images of Aplp1 FL, ΔE1, ΔAcD+E2, ΔJMR, ΔTM, ΔICD, ΔGFLD, ΔCuBD with WGA-488. **d**, The anti-Myc immunostaining images of Lag3 FL, ΔD1, ΔD2, ΔD3, ΔD4, ΔTM, ΔICD, using membrane marker wheat germ agglutinin (WGA) conjugated with Alexa Fluor^{TM} 488. **e**, The detailed information for the expression on membrane. **f**, Live images of the endocytosis of α-syn-pHrodo PFF. α-Syn PFF were conjugated with a pH-dependent dye (pHrodo red), in which fluorescence increases as pH decreases from neutral to acidic environments. Four groups include wildtype (WT) neurons, WT neurons overexpressing Aplp1 by lentivirus transduction (WT + Aplp1), *Aplp1*$^{-/-}$ neurons, and Aplp1$^{-/-}$ neurons with Aplp1 overexpression (*Aplp1*$^{-/-}$ + Aplp1). Scale bar, 20 μm. **g**, The co-localization of internalized α-syn-biotin PFF (red), Rab7 (green) and FLAG-Aplp1 (grey scale) in soma of WT and Aplp1$^{-/-}$ neuronal culture was assessed by means of confocal microscopy. Scale bar, 10 μm. Source data are provided as a Source Data file.

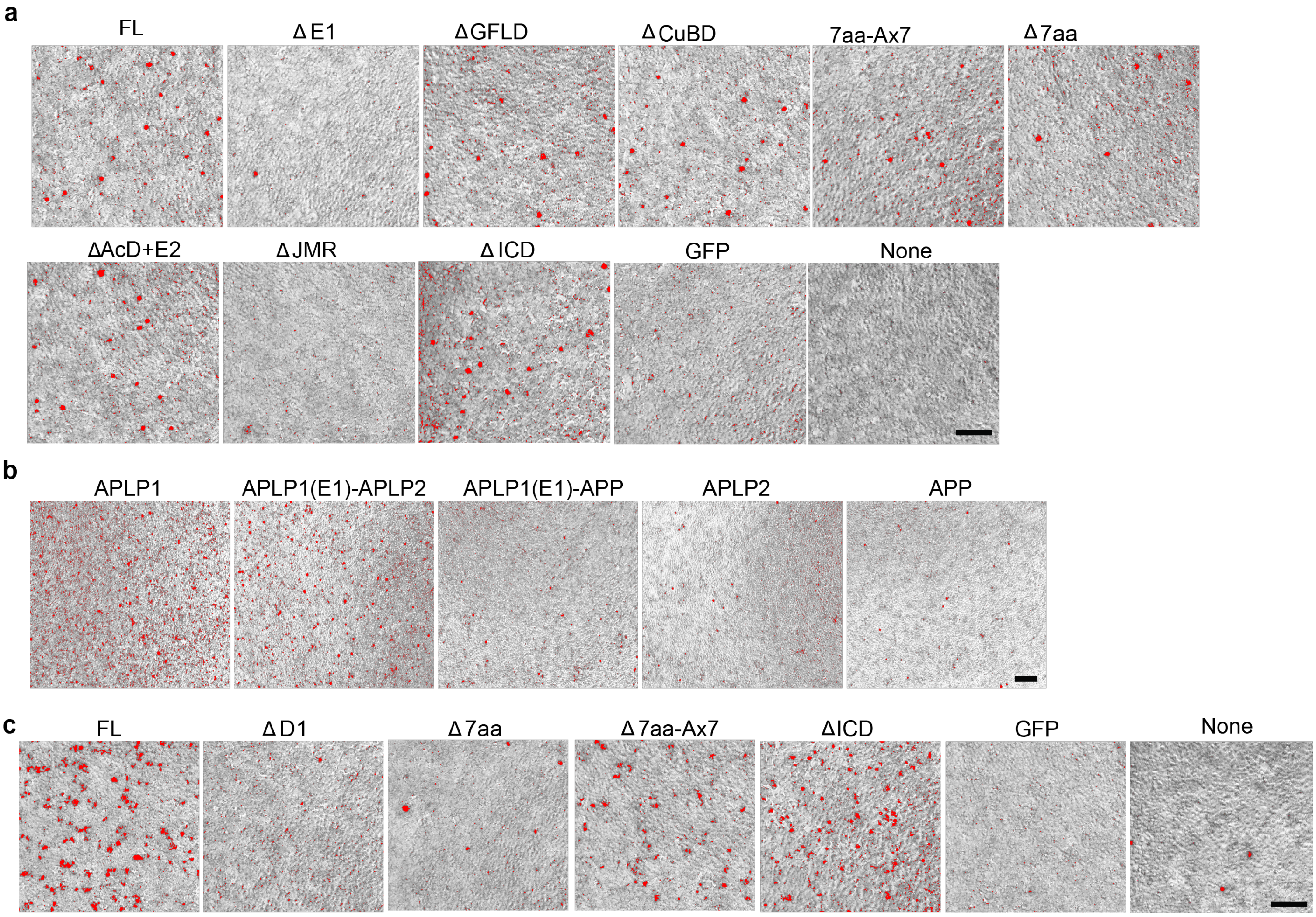

Supplementary Fig. 2 | α-Syn PFF binds to Aplp1 in cell surface binding assay. a, Representative images of binding signals of deletion mutants of Aplp1 with α-syn-biotin PFF. **b,** Representative images of binding of Aplp1(E1)-Aplp2 and Aplp1(E1)-App chimeras with α-syn-biotin PFF. **c**, Representative images of binding of deletion mutants of Lag3 with α-syn-biotin PFF. Binding signal is represented in red. n= 3 independent experiments, Scale bar, 100 μm. Source data are provided as a Source Data file.

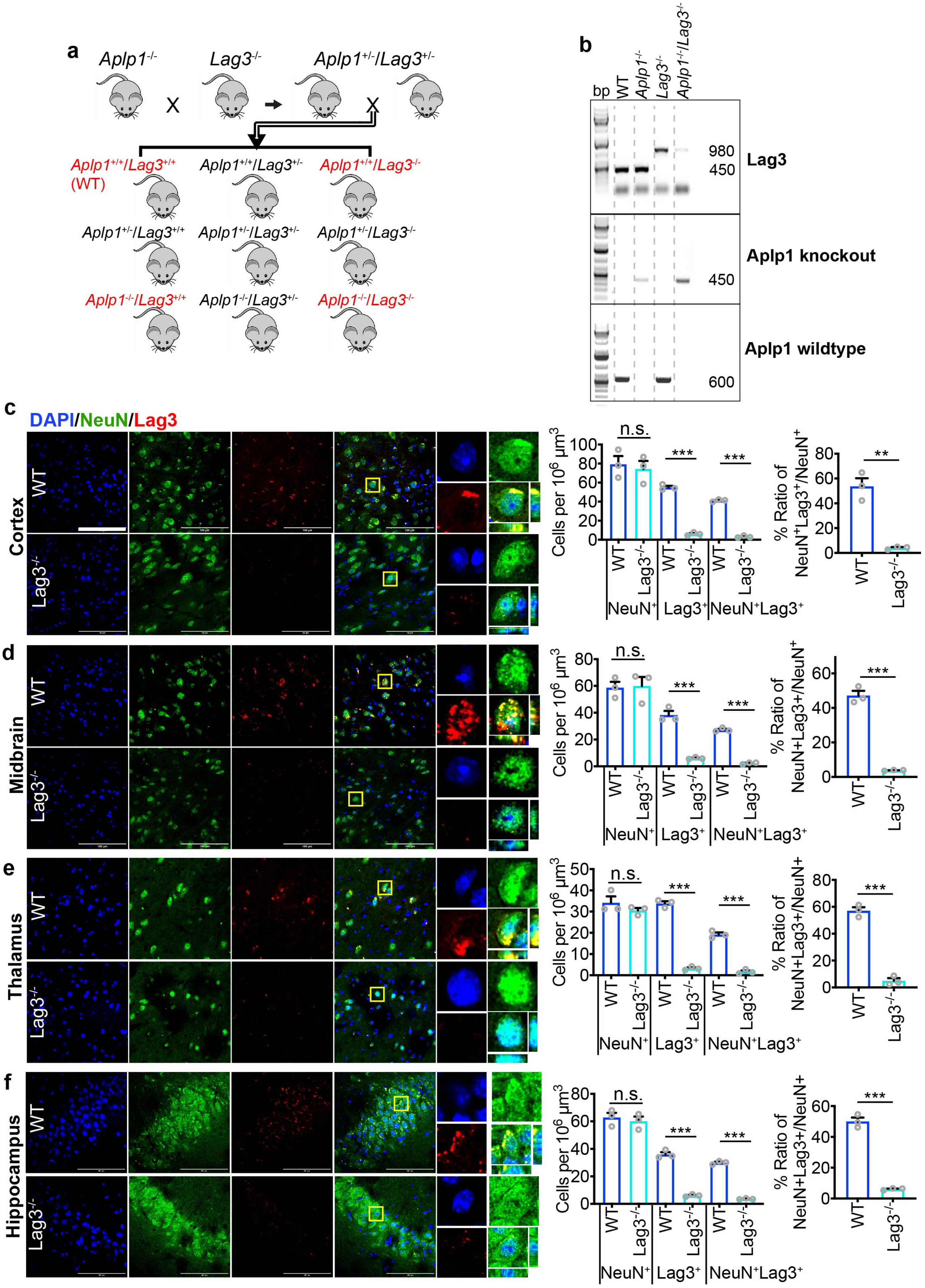

a
Aplp1-/-
Lag3-/-
Aplp1+/-/Lag3+/-
Aplp1+/+/Lag3+/+ (WT)
Aplp1+/+/Lag3+/-
Aplp1+/+/Lag3-/-
Aplp1+/-/Lag3+/+
Aplp1+/-/Lag3+/-
Aplp1+/-/Lag3-/-
Aplp1-/-/Lag3+/+
Aplp1-/-/Lag3+/-
Aplp1-/-/Lag3-/-
b
bp
WT
Aplp1-/-
Lag3-/-
Aplp1-/-/Lag3-/-
980
450
Lag3
Aplp1 knockout
450
Aplp1 wildtype
600
c
DAPI/NeuN/Lag3
Cortex
WT
Lag3-/-
Cells per 10⁶ μm³
n.s.
***
% Ratio of NeuN+Lag3+/NeuN+
**
NeuN+
Lag3+
NeuN+Lag3+
d
Midbrain
e
Thalamus
f
Hippocampus

Supplementary Fig. 3 | Lag3 is expressed in neurons in WT mice. a, Breeding strategy of the double knockout of *Aplp*1 and *Lag3* (*Aplp1*$^{-/-}$/*Lag3*$^{-/-}$) mice. **b**, Genotyping confirmation of WT, *Aplp1*$^{-/-}$, *Lag3*$^{-/-}$ and *Aplp1*$^{-/-}$/*Lag3*$^{-/-}$ mice. **c**,**d**,**e**,**f**, Co-Immunostaining of anti-Lag3 and anti-NeuN in four brain regions of WT and *Aplp1*$^{-/-}$/*Lag3*$^{-/-}$ mice: the cortex **(c)**, the midbrain **(d)**, the thalamus **(e)**, and the hippocampus **(f)**. Scale bar, 100 µm. Data are the means ± SEM. Each scatter dot represents one image taken and the analysis was obtained from 3 WT mice and 3 *Lag3*$^{-/-}$ mice. Student's *t*-test, ****P* < 0.001. n.s., not significant. Source data are provided as a Source Data file.

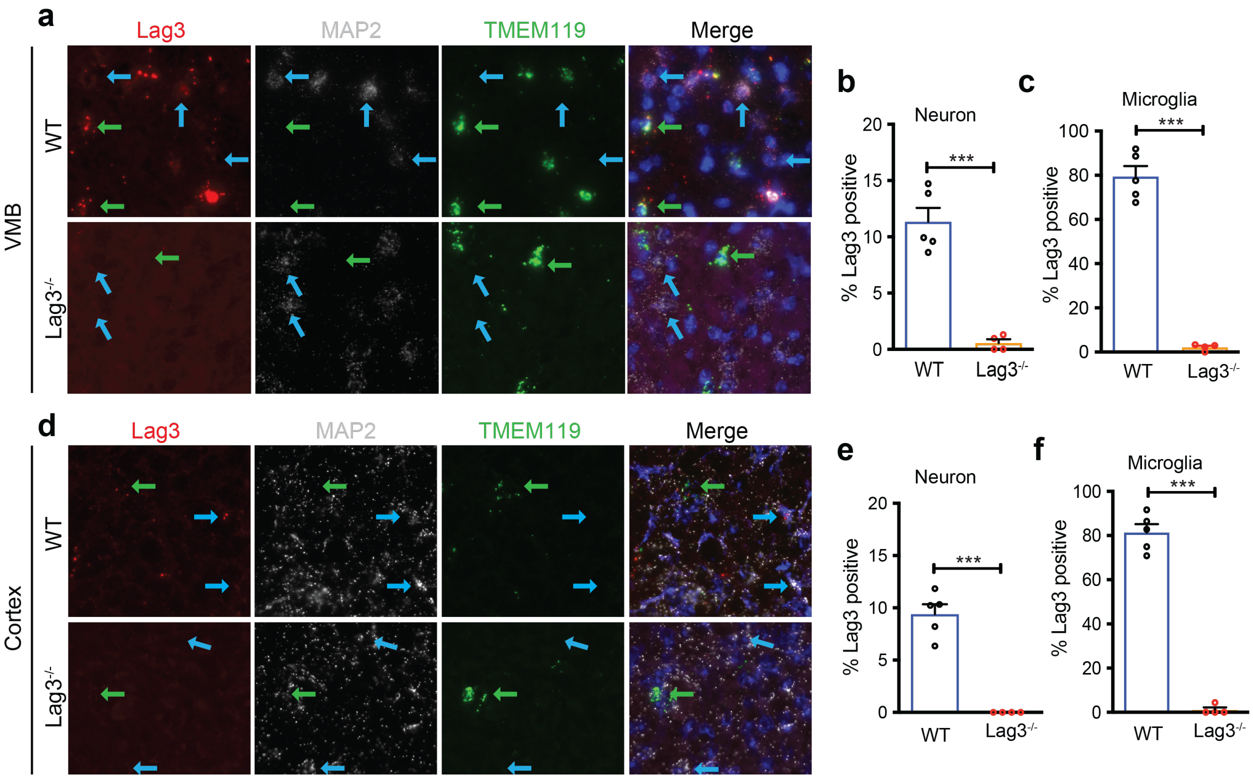

Supplementary Fig. 4 | Lag3 is detected in the neurons by RNAscope.: **a)** Co-localization of Lag3 (red) inside neurons labelled by MAP2 (white) and microglia labelled by TMEM119 (green) in VMB region in WT and Lag3$^{-/-}$ mice. **b-c)** quantification of Lag3 positive cells in neurons (**b**) and in microglia (**c**) N = 5 for WT mice and N = 4 for *Lag3*$^{-/-}$ mice, At least 200 neurons were counted per mice. d) Co-localization of Lag3 (red) inside neurons labelled by MAP2 (white) and microglia labelled by TMEM119 (green) in cortex in WT and *Lag3*$^{-/-}$ mice. **e-f**) quantification of Lag3 positive cells in neurons (**e**) and in microglia (**f**) N = 5 for WT and N = 4 for Lag3$^{-/-}$ mice, At least 200 neurons were counted per mice. blue arrow indicates neuron and green arrow indicates microglia. Data are the means ± SEM, two tailed Student *t*-test, ***P < 0.001. Source data are provided as a Source Data file.

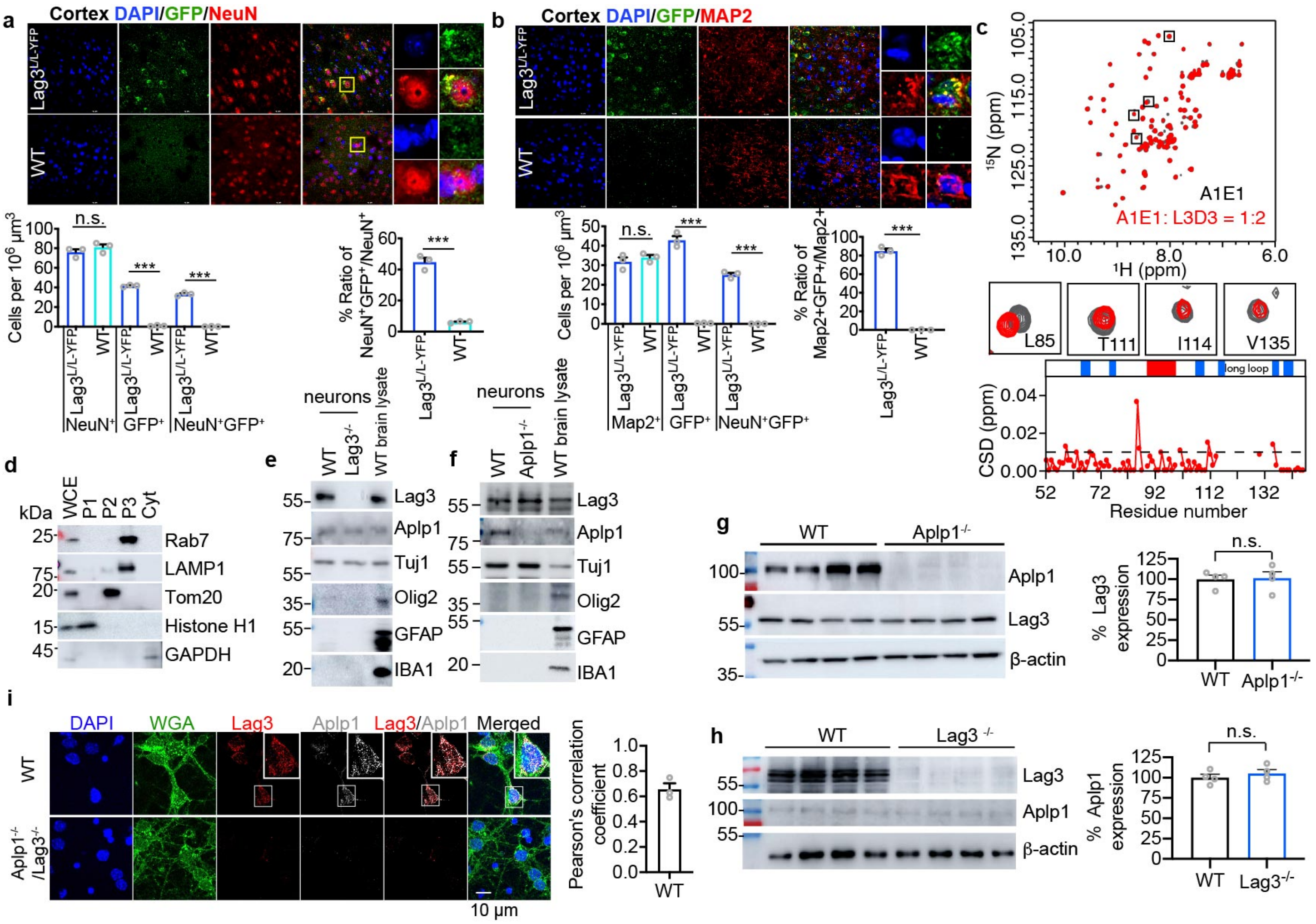

Supplementary Fig. 5 | Lag3 is expressed in neurons in *Lag3*$^{L/L\text{-}YFP}$ mice. a,**b**, Cellular localization of Lag3 in a Lag3 Loxp reporter line with a YFP (yellow fluorescence protein) signal knocked into the Lag3 locus (*Lag3*$^{L/L\text{-}YFP}$). The immunoreactivity of anti-GFP (Lag3), which recognizes YFP co-localized with immunostainings of anti-NeuN **(a)** and anti-MAP2 **(b)** in the cortex. Scale bar, 100 μm. Data are the means ± SEM. Each scatter dot represents one image taken and the analysis was obtained from 3 *Lag3*$^{L/L\text{-}YFP}$ mice and 3 WT mice. Student's *t*-test, ****P* < 0.001. n.s., not significant. **c**, A1E1 interacts with L3D3. (Upper inset): Overlay of the 2D ^{1}H-^{15}N HSQC spectra of A1E1 alone (black) and in the presence of 2 molar folds of L3D3 (red). (Middle inset): The same 4 residues that with significant CSDs (> 0.03 ppm) upon L3D2 titration are highlighted in the black boxes and zoomed in. Histogram of the CSDs of A1E1 in the presence of L3D3 at a molar ratio (A1E1/L3D3) of 1:2. (Bottom inset): The domain organization of A1E1 is indicated on the top, with blue boxes indicating the β-strands and the red box indicating the α-helix. A dashed line was drawn to highlight the residues with CSDs > 0.01 ppm. **d**, Validation of the enrichment of the endolysosomes in the P3 fraction. The centrifuged pellets (P1, P2, and P3) of each step and the rest cytosol (Cyt) were immunoblotted with Rab7, LAMP1, Tom20, Histone H1, and GAPDH. P3 is the target fraction sample for endolysosome enrichment. **e**,**f**, Validation of WT

primary cortical neurons for Lag3 expression and Aplp1 expression with Tuj1, Oligo2, GFAP, IBA1 immunoblotting. $Lag3^{-/-}$ and $Aplp1^{-/-}$ primary cortical neurons are the negative controls, and WT brain lysate is the positive control. **g**, No significant difference of Lag3 expression between WT and $Aplp1^{-/-}$ brain lysate with immunoblots. Data are the means ± SEM, Student's *t*-test; n.s., not significant. **h**, No significant difference of Aplp1 expression between WT and $Lag3^{-/-}$ brain lysate with immunoblots. Data are the means ± SEM, Student's *t*-test; n.s., not significant. **i**, The co-staining of WGA (plasma membrane maker), Lag3, and Aplp1 in non-permeabilized WT and $Aplp1^{-/-}/Lag3^{-/-}$ primary cortical neurons. The data was obtained from 27 neurons in 3 independent cultures. The Pearson's correlation coefficient is 0.62 > 0.5. Scale bar, 5 μm. Source data are provided as a Source Data file.

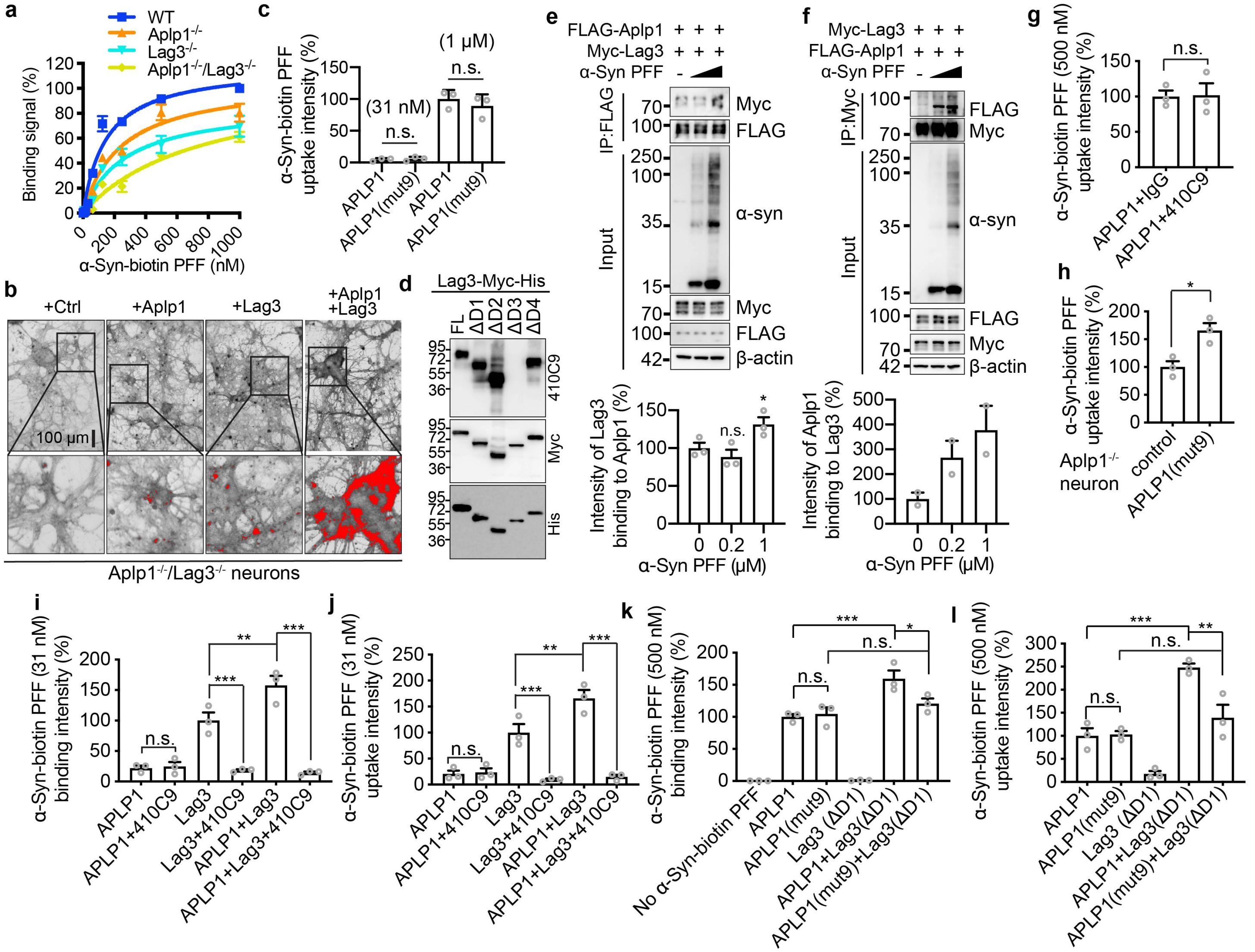

Supplementary Fig. 6 | α-syn PFF binding to Lag3 and Aplp1. a, Binding signal of α-syn-biotin PFF to WT, *Aplp1*$^{-/-}$, *Lag3*$^{-/-}$, and *Aplp1*$^{-/-}$/*Lag3*$^{-/-}$ cortical neurons, normalized by the total cell numbers. WT-K_d=149 nM, *Aplp1*$^{-/-}$-K_d=245 nM, *Lag3*$^{-/-}$-K_d=266 nM, *Aplp1*$^{-/-}$/*Lag3*$^{-/-}$-K_d=767 nM. Data are the means ± SEM, N = 3 independent experiments. **b**, Binding images of α-syn-biotin PFF (63 nM) to *Aplp1*$^{-/-}$/*Lag3*$^{-/-}$ neurons transduced with Aplp1, Lag3, or Aplp1+Lag3 by lentivirus, normalized by the total cell numbers. The binding intensity (red signal) was extracted with the ImageJ: Image/Adjust/Threshold setting value 80. Scale bar, 100 μm. **c**, The uptake signal of α-syn-biotin PFF (31 nM and 1 μM) in Aplp1 nor Aplp1(mut9) transfected cells. **d**, Epitope of anti-Lag3 410C9 for mouse Lag3. Deletion mutants of Lag3 with Myc and His tags, were transfected in HEK293FT cells, and the cell lysis was probed with 410C9, anti-Myc and anti-His antibodies. Anti-Lag3 410C9 can distinguish the D3 domain of mouse Lag3. **e**, α-Syn PFF treatment of the cellular extract of FLAG-Aplp1 and Myc-Lag3 transfected HEK293FT cells significantly increases the co-IP of FLAG-Aplp1 and Myc-Lag3. Data are the means ±

SEM, ***$P < 0.001$, n.s., not significant; one-way ANOVA followed by Tukey's correction. $N = 3$ independent experiments. **f**, α-Syn PFF increases the co-IP of Myc-Lag3 and FLAG-Aplp1. $N = 2$ independent experiments. **g**, Anti-Lag3 (410C9) cannot significantly inhibit the uptake intensity of α-syn-biotin PFF in APLP1-expressing cells. Data are the means ± SEM, Student's *t*-test; n.s., not significant. **h**, APLP1(mut9) can significantly increase the uptake intensity of α-syn-biotin PFF in Aplp1$^{-/-}$ primary cortical neurons. Data are the means ± SEM, Student's *t*-test; $P < 0.05$. **i**, The binding intensity of α-syn-biotin PFF (31 nM) to APLP1, Lag3, and APLP1-Lag3, and the inhibitory efficacy of 410C9. **j**, The uptake intensity of α-syn-biotin PFF (31 nM) by APLP1, Lag3, and APLP1-Lag3 , and the inhibitory efficacy of 410C9. **k**,**l**, APLP1 and Lag3(ΔD1) can increase binding **(k)** and uptake **(l)** of α-syn-biotin PFF (500 nM), compared to APLP1 alone. APLP1(mut9) significantly reduced the binding and uptake of α-syn-biotin PFF with Lag3(ΔD1), and there is no significant difference between APLP1(mut9) and APLP1(mut9)-Lag3(ΔD1). **i**,**j**,**k**,**l**, Statistical significance was determined by using one-way ANOVA followed with Sidak's correction, *$P < 0.05$, **$P < 0.01$, ***$P < 0.001$, n.s., not significant. Source data are provided as a Source Data file.

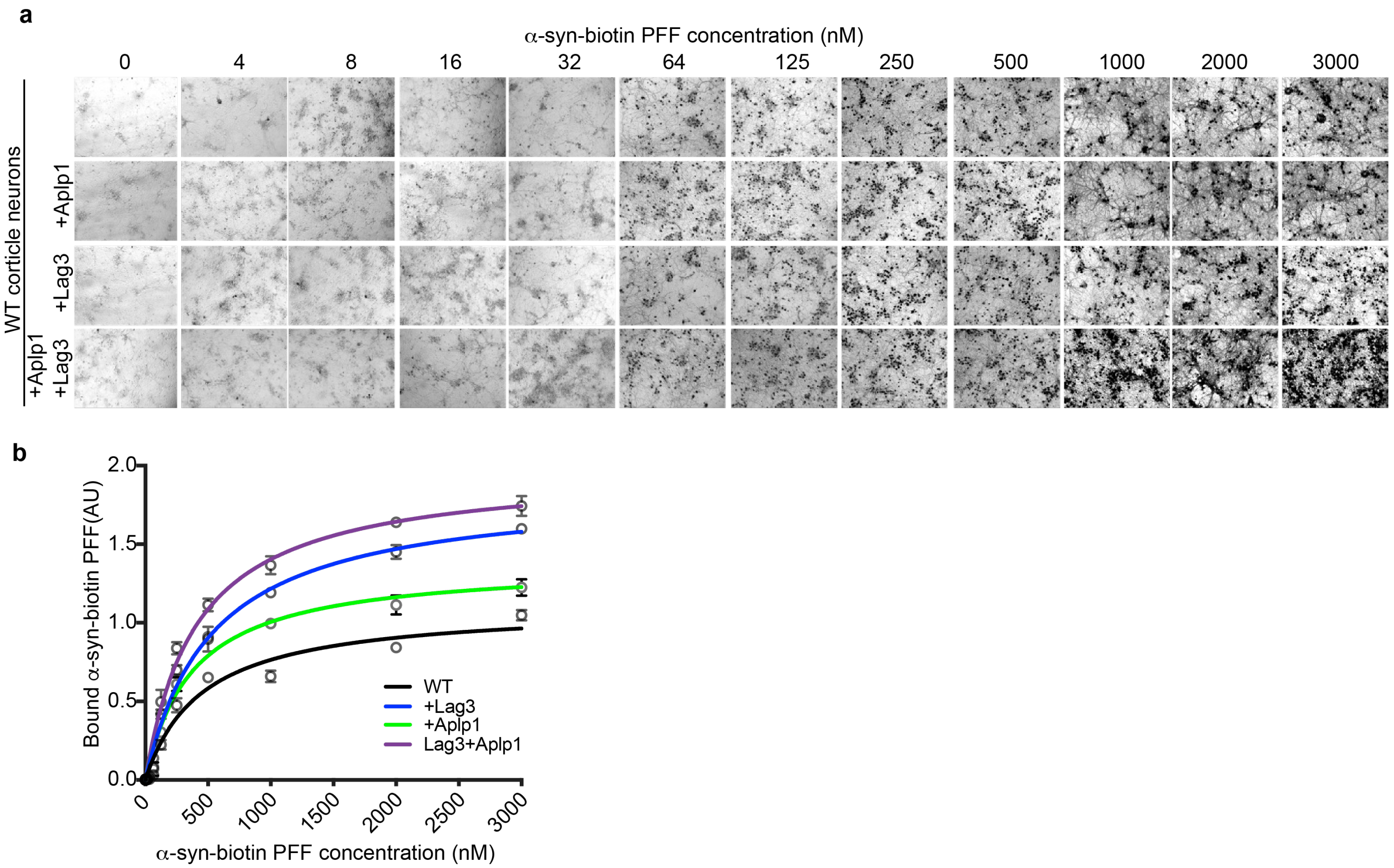

Supplementary Fig. 7 | α-Syn PFF binding to primary neurons overexpressing Aplp1 and Lag3. **a**, Representative images of α-Syn PFF binding to WT primary cortical neurons and neurons overexpressing Aplp1, Lag3 or Aplp1 and Lag3. **b**, Quantification of α-syn-biotin PFF binding signals to WT, Aplp1, Lag3 overexpressing primary cortical neurons, normalized to WT binding signal (*N* = 3). Source data are provided as a Source Data file.

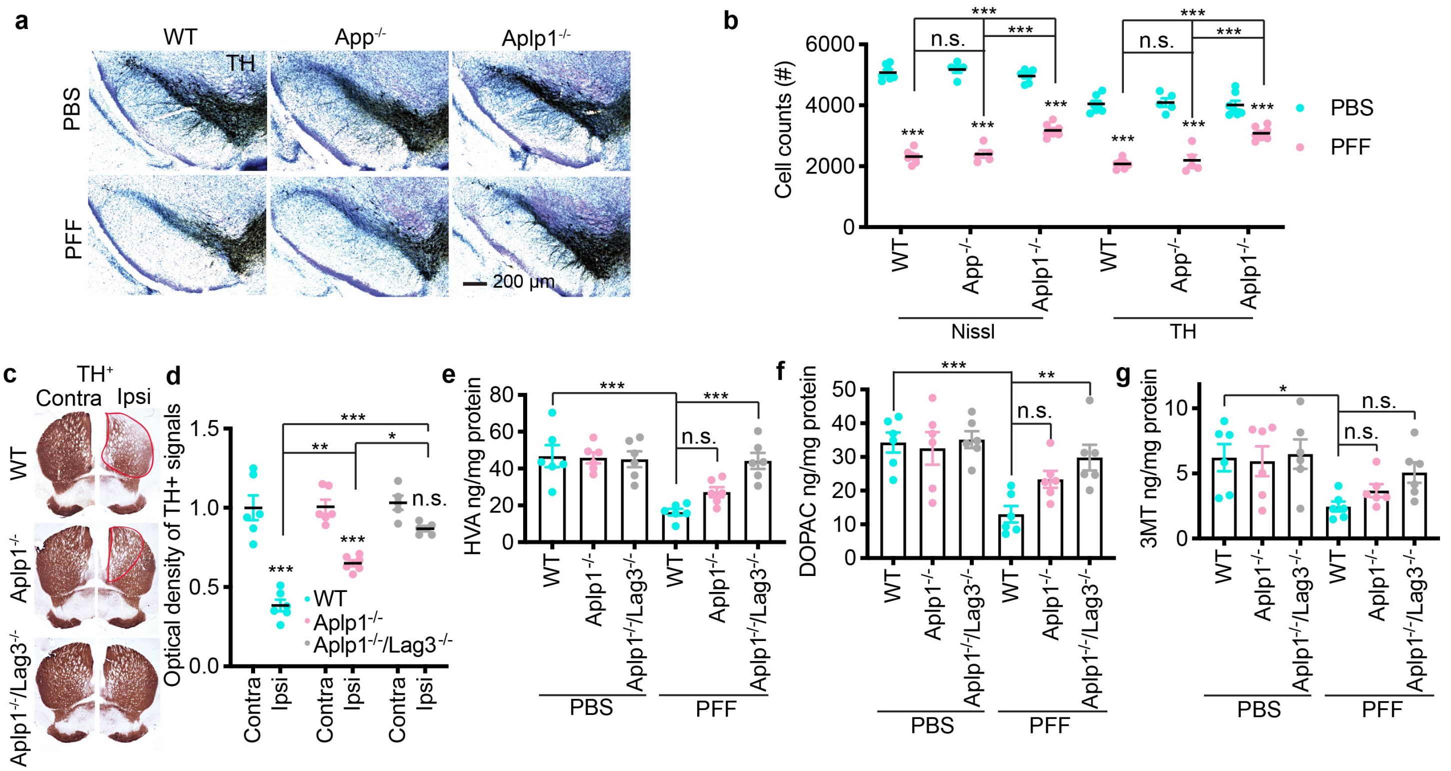

Supplementary Fig. 8 | Deletion of Aplp1 and the Aplp1-Lag3 prevent neurodegeneration induced by α-syn PFF. a, Representative TH and Nissl staining images in the SNpc of α-syn PFF-injected hemisphere in the WT, $App^{-/-}$, and $Aplp1^{-/-}$ (WT: N = 7; $App^{-/-}$: N = 5; $Aplp1^{-/-}$: N = 7). **b**, Stereological counting of the number of Nissl- and TH-positive neurons in the substantia nigra via unbiased stereological analysis after 6 months of α-syn PFF injection in the WT, $App^{-/-}$, and $Aplp1^{-/-}$ mice. Data are the means ± SEM. Statistical significance was determined by using one-way ANOVA followed with Tukey's correction; ***P < 0.001, n.s., not significant. **c**, Representative TH immunohistochemistry images in the striatum of α-syn PFF injected brain of WT, $Aplp1^{-/-}$, and $Aplp1^{-/-}/Lag3^{-/-}$ mice. **d**, Quantifications of TH-immunopositive fiber densities in the striatum (WT: N = 6; $Aplp1^{-/-}$: N = 6; $Aplp1^{-/-}/Lag3^{-/-}$: N = 5). Data are the means ± SEM. Statistical significance was determined by using one-way ANOVA followed with Tukey's correction, *P < 0.05, **P < 0.01, ***P < 0.001, n.s., not significant. **e**,**f**,**g**, Striatal metabolites levels in WT, $Aplp1^{-/-}$, and $Aplp1^{-/-}/Lag3^{-/-}$ mice, were measured at 180 days post-injection by HPLC. Data are the means ± SEM, N = 6 mice per group, one-way ANOVA with Dunnett's correction. Source data are provided as a Source Data file.

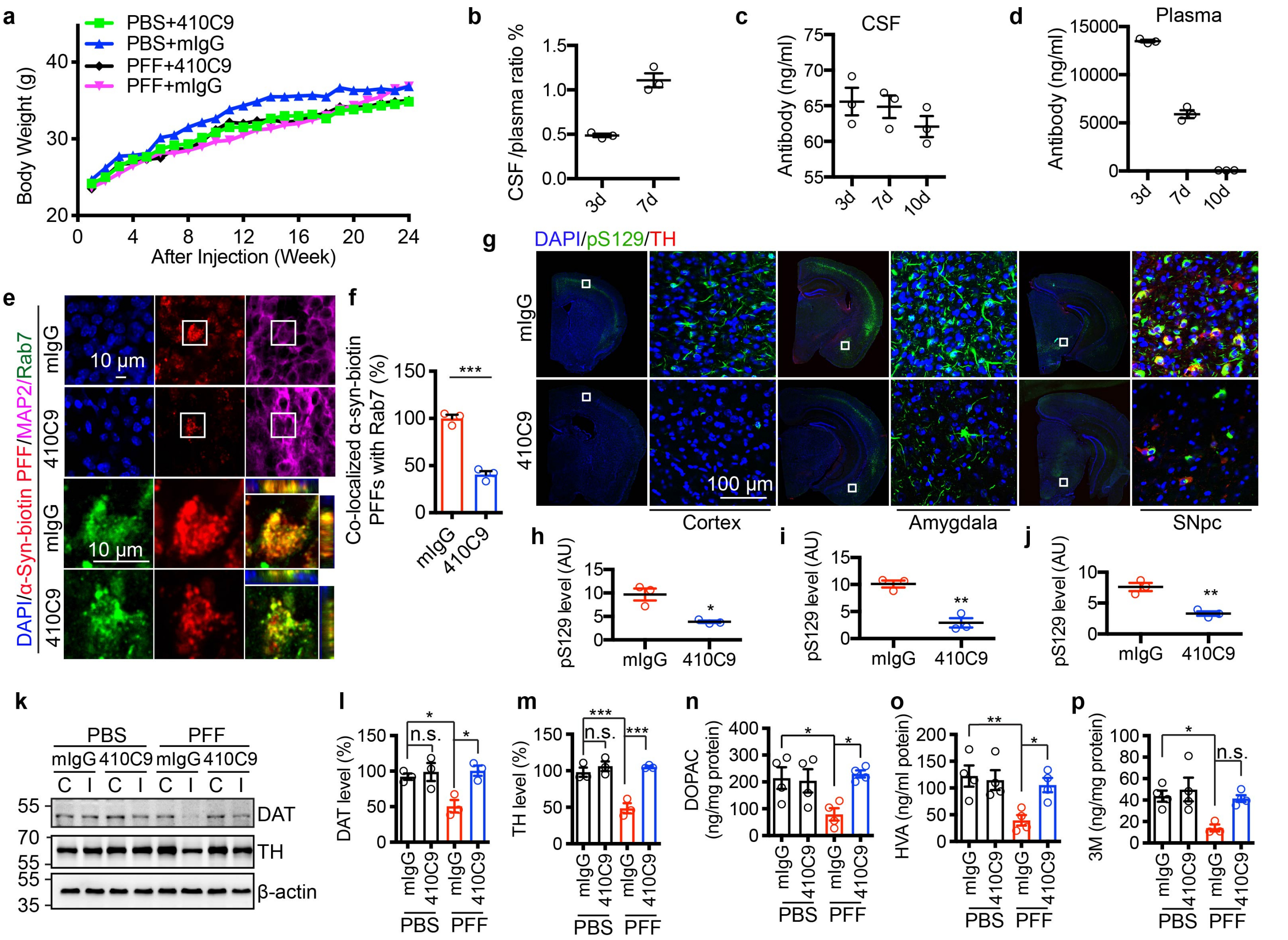

Supplementary Fig. 9 | Anti-Lag3 410C9 blocks neurodegeneration induced by α-syn PFF *in vivo*. **a**, Body weight assessment of WT mice for 6-months after stereotaxic injection of α-syn PFF or PBS, and i.p.(intraperitoneal) treated with 410C9 or mIgG. **b**, Ratio of 410C9 detected in CSF vs plasma was roughly 0.5% at 3 days. *N* = 3 mice per time points. Values are means ± SEM. **c**,**d**, 410C9 turnover in WT mice. At 3 days, 7 days, 10 days following a single i.p. injection of 410C9 (10 mg/kg), WT mice (without α-syn PFF treatment) were sacrificed; their **(c)** CSF and **(d)** plasma were collected to determine the level of circulating 410C9 by labeling with a near infrared dye (IR) by plate reader. **e**,**f**, Co-localization of α-syn-biotin PFF with Rab7 is inhibited by 410C9 *in vivo*. The signal of α-syn-biotin PFF colocalizes with Rab7 in neurons (MAP2 staining) in the striatum of WT mice. Intrastriatal injection of α-syn-biotin PFF were performed 3 days after i.p. injection of mIgG or 410C9 (10 mg/kg) in WT mice. Scale bar 10 μm. Data are the means ± SEM, *N* = 3 mice per group, mIgG (63 cells) and 410C9 (112 cells). Statistical significance was determined by using Student's *t*-test; ***P < 0.001. **g**,**h**,**i**,**j**, The representative images of pS129 immunostaining from α-syn PFF-injected WT mice with treatment of 410C9 or mIgG. α-Syn PFF-induced pathology is reduced by 410C9. Quantification of pS129 intensity

in **(h)** cortex, **(i)** amygdala, and **(j)** SNpc region. Bars represent mean ± SEM, $N = 3$ mice per group. Statistical significance was determined by using Student's *t*-test; $^{*}P < 0.05$, $^{**}P < 0.01$. **k**,**l**,**m**, Immunoblot analysis of the striatum from α-syn PFF or PBS in WT mice, using antibodies against DAT (dopamine transporter) and TH. Mean intensity values are the ratios of the ipsilateral (I) injected side to contralateral (C) side for DAT and TH. Data are the means ± SEM, $N = 3$ mice per group, one-way ANOVA with Dunnett's correction. $^{*}P < 0.05$, $^{***}P < 0.001$, n.s., not significant. **n**,**o**,**p**, Striatal metabolites levels of DOPAC, HVA, and 3M were measured by HPLC-ECD. Data are the means ± SEM, $N = 4$ mice per group, one-way ANOVA with Tukey's correction; $^{*}P < 0.05$, $^{**}P < 0.01$, n.s., not significant. Source data are provided as a Source Data file.

Supplementary Table 1: List of Oligonucleotides used in this study

Oligonucleotides (Primers)	Sequence (5’ > 3’)
To add Glycine rich linker between Lag3 and myc tag (by the Ligation method)	
Linker oligo sense	agcttggcggcggaggatcgggtggtagtggtggttcac
Linker oligo antisense	agctgtgaaccaccactaccacccgatcctccgccgcca
For making Lag3 deletion mutants (by the Ligation method)	
mouseLag3 del ICD For	atatatctcgagatgagggaggacctgc
MouseLag3 del ICD Rev	cgcgaagctttcttctcagtagcaactgtt
MouseLag3 del CP-TM For1	atatatctcgagatgagggaggacctgc
MouseLag3 del CP-TM Rev1	atatatgctagcctcgtacagctgacactgc
MouseLag3 del CP-TM For2	atatatgctagcttttctgccttagaacatggg
MouseLag3 del CP-TM Rev2	atatataagcttgagctgcctgggctct
For making Lag3 7aa substitution or deletion mutants (by the inFusion method)	
mLag3 7aa to A sub For1	gccctctagactcgagatgaggg
mLag3 7aa to A sub Rev1	tgctgcggccgctgctgctggagccacgctcagcac
mLag3 7aa to A sub For2	gcagcggccgcagcagcacagcccctgcatccccac
mLag3 7aa to A sub Rev2	gttcgggcccaagcttgagc
mLag3 7aa del For1	gccctctagactcgagatgaggg
mLag3 7aa del Rev1	ggggctgtggagccacgctcagcac
mLag3 7aa del For2	tggctccacagcccctgcatccccac
mLag3 7aa del Rev2	gttcgggcccaagcttgagc
For making Aplp1 deletion mutants (by the inFusion method)	
MouseAplp1 del JMR For1	acccaagctggctagaccatgtctgcacttctgatc
MouseAplp1 del JMR Rev1	aaggcctcgagcagccccaggctctg
MouseAplp1 del JMR For2	gctgctcgaggccttgtcaggtctgc
MouseAplp1 del JMR Rev2	ccgtttaaacgctagtcaaggtcgttcttccaggaag
MouseAplp1 del TM For1	acccaagctggctagaccatgtctgcacttctgatc
MouseAplp1 del TM Rev1	ttgcgcagtcgggacactccagtccc
MouseAplp1 del TM For2	gtcccgactgcgcaagaagaaaccctа
MouseAplp1 del TM Rev2	ccgtttaaacgctagtcaaggtcgttcttccaggaag
MouseAplp1 del GFLD For1	acccaagctggctagaccatgtctgcacttctgatc
MouseAplp1 del GFLD Rev1	cgacagccgcgcccaagcttgtcgtc
MouseAplp1 del GFLD For2	tgggcgcggctgtcggttcttgcac
MouseAplp1 del GFLD Rev2	ccgtttaaacgctagtcaaggtcgttcttccaggaag
MouseAplp1 del CuBD For1	acccaagctggctagaccatgtctgcacttctgatc
MouseAplp1 del CuBD Rev1	gcaggaggttcgggcactagcaggg
MouseAplp1 del CuBD For2	gcccgaacctcctgcaactcccaacc
MouseAplp1 del CuBD Rev2	ccgtttaaacgctagtcaaggtcgttcttccaggaag
For making Aplp1 7aa substitution or deletion mutants (by the inFusion method)	
mAplp1 7aa to A sub For1	acccaagctggctagaccatgtctgcacttctgatc
mAplp1 7aa to A sub Rev1	tgctgcggccgctgctgcacaccagcgctccatcgg
mAplp1 7aa to A sub For1	gcagcggccgcagcagcatgcgcccacccccacca
mAplp1 7aa to A sub Rev2	ccgtttaaacgctagtcaaggtcgttcttccaggaag
mAplp1 7aa del For1	acccaagctggctagaccatgtctgcacttctgatc
mAplp1 7aa del Rev1	gggcgcaacaccagcgctccatcg
mAplp1 7aa del For2	gctggtgttgcgcccacccccacca
mAplp1 7aa del Rev2	ccgtttaaacgctagtcaaggtcgttcttccaggaag
For making Aplp1-E1ACD - Aplp2/App chimera (by the inFusion method)	
Aplp1-Aplp2 For1	acccaagctggctagaccatgtctgcacttctgatc
Aplp1-Aplp2 Rev1	agcagacgactctgctaaccatgacagg
Aplp1-Aplp2 For2	gcagagtcgtctgctcccaggaggcc
Aplp1-Aplp2 Rev2	ccgtttaaacgctagttaaatctgcatctgctccagg

Aplp1-App For1	acccaagctggctagaccatgtctgcacttctgatc
Aplp1-App Rev1	cagagcagactctgctaaccatgacagg
Aplp1-App For2	gcagagtctgctctgaacaagccgag
Aplp1-App Rev2	ccgtttaaacgctagttagttctgcatttgctcaaag
For making FUGW-mouse Lag3 or mouse Aplp1 viral vector	
cFUGW mouseLag3 For1	cgactctagaggatccaccatgagggaggacctgctccttg
cFUGW mouseLag3 Rev1	gcttgatatcgaattctcagagctgcctgggctctg
cFUGW mouesLag3-myc R	gcttgatatcgaattctcacagatcctcttctgagatgag
cFUGW mouseAplp1 For1	cgactctagaggatcgccaccatgtctgcacttctgatc
cFUGW mouseAplp1 Rev1	gcttgatatcgaatttcaaggtcgttcttccaggaag
qPCR primers	
Mouse Lag3 For (set1)	tccgcctgcgcgtcg
Mouse Lag3 Rev (set1)	gacccaatcagacagcttgaggac
Mouse Lag3 For (set2)	ttgcttctgggactgctttg
Mouse Lag3 Rev (set2)	gccactgtctggttgatgttg
Mouse beta actin For	ggctgtattcccctccatcg
Moues beta actin Rev	ccagttggtaacaatgccatgt
For making FLAG-Aplp1(mut9)	
NheI-Flg-hAPLP1-F:	ctagctagcatggactacaaggatgatgacgacaaagggcccgccagc
XhoI-His-hAPLP1-R:	ccgctcgagtcaatggtgatggtggtgatggggtcgttcctccaggaa

Supplementary Table 2: List of antibodies used in this study:

Reagent or Resource	Source	Identifier
**Antibodies**		
Mouse anti-lymphocyte-activation gene 3 (Lag3)	Generated in Dario Vignali lab and Millipore Sigma	410C9 (4-10-C9), MABF954, RRID: N/A
Rabbit anti-amyloid β precursor like protein 1 (Aplp1)	Generated in Gopal Thinakaran lab	A1NT, RRID: N/A
		CT11, RRID: N/A
Mouse anti-α-synuclein	BD Bioscience	Cat# 610787, RRID:AB_398108
Rabbit anti-pS129-α-synuclein	Abcam	Cat# ab51253, RRID:AB_869973
Mouse anti-neuronal nuclei (NeuN)	Millipore Sigma	Cat# MAB377, RRID:AB_2298772
Rabbit anti-Tyrosine Hydroxylase (TH)	Novus Biologicals	Cat# NB300-109, RRID:AB_10077691
Mouse anti-Myc-HRP	Cell Signaling Technology	Cat# 2040S, RRID:AB_2148465
Rabbit anti-Rab5	Abcam	Cat# ab18211, RRID:AB_470264
Rabbit anti-Rab7	Cell Signaling Technology	Cat# 2094, RRID:AB_2300652
Rabbit anti-lysosomal associated membrane protein 1 (LAMP1)	Abcam	Cat# ab24170, RRID:AB_775978
Rabbit anti-Clathrin	Abcam	Cat# ab59710, RRID:AB_941047
Rabbit anti-Dynamin II	Abcam	Cat# ab3457, RRID:AB_2093679
Rabbit anti-Caveolin-1 (CAV-1)	Abcam	Cat# ab2910, RRID:AB_303405
Mouse anti-β-actin	ThermoFisher Scientific	Cat# MA5-15739-HRP, RRID: AB_2537667
Mouse anti-microtubule-associated protein 2 (MAP2)<br>Rabbit anti-MAP2	Sigma<br>Millipore Sigma	Cat# M9942, RRID:AB_477256<br>Cat# AB5622, RRID:AB_91939
Rabbit anti-ionized calcium binding adaptor molecule 1 (IBA1)	Wako	Cat# 019-19741, RRID: AB_839504
Rabbit anti-glial fibrillary acidic protein (GFAP)	Abcam	Cat# ab7260, RRID:AB_305808
Mouse anti-GFP	ThermoFisher Scientific	Cat# MA5-15256, RRID: AB_10979281
Donkey Anti-Rabbit IgG, Whole Ab ECL Antibody, HRP Conjugated	GE Healthcare	Cat#NA934; RRID: AB_772206
Sheep Anti-Mouse IgG, Whole Ab ECL Antibody, HRP Conjugated	GE Healthcare	Cat#NA931; RRID: AB_772210

Uncropped Blots:

Figure 2b

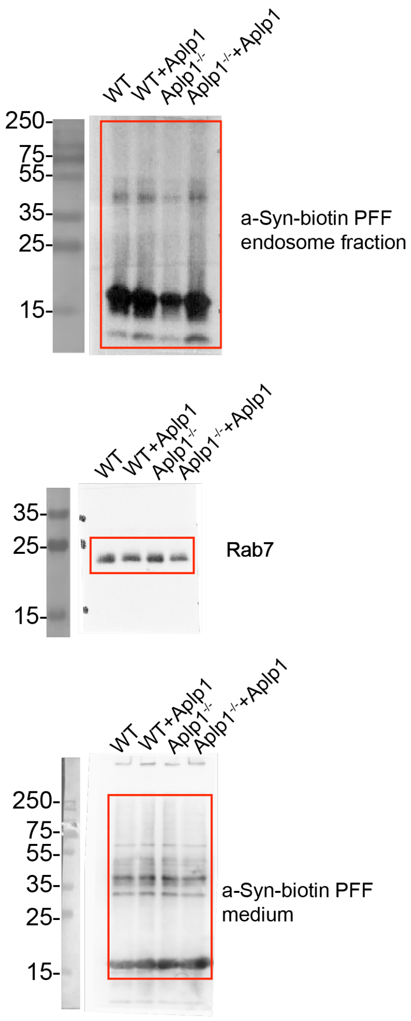

Figure 2i

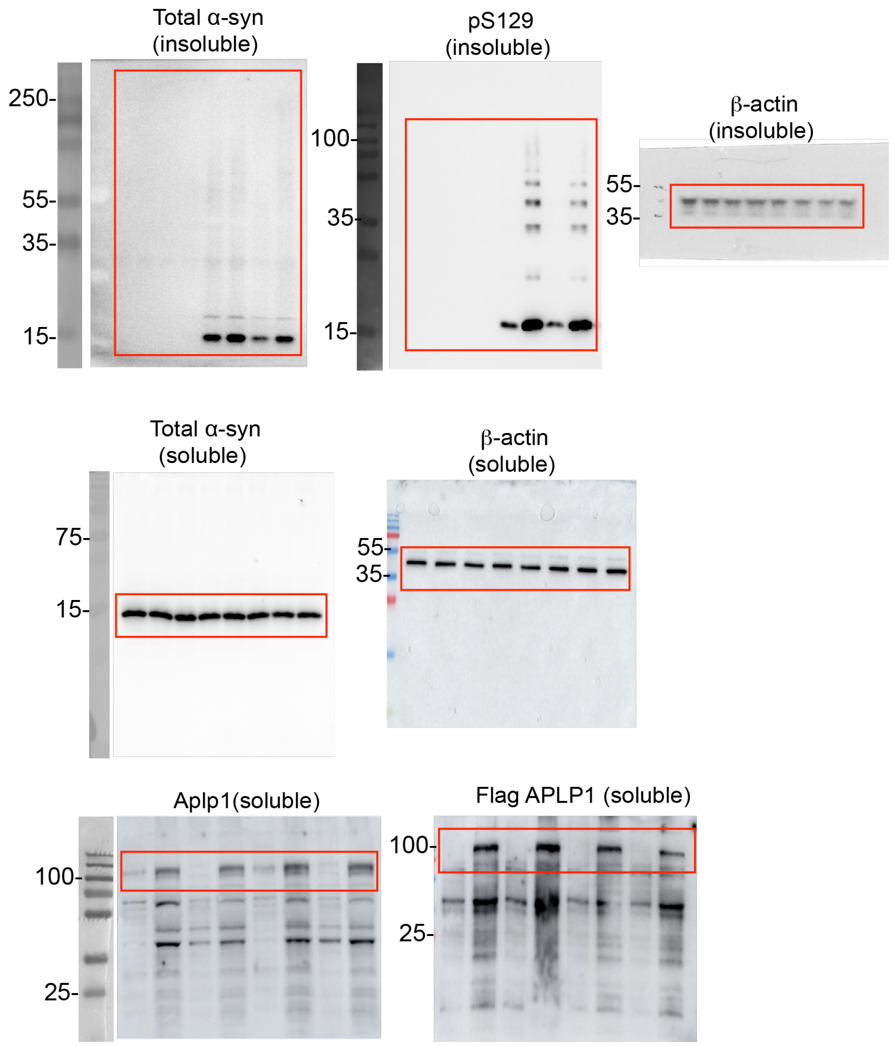

Figure 3a

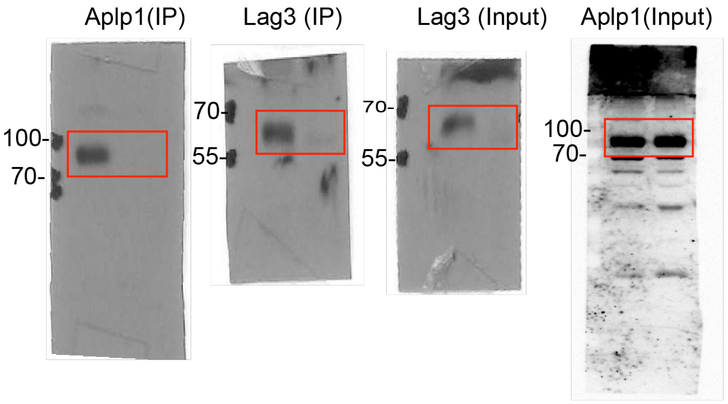

Figure 3b

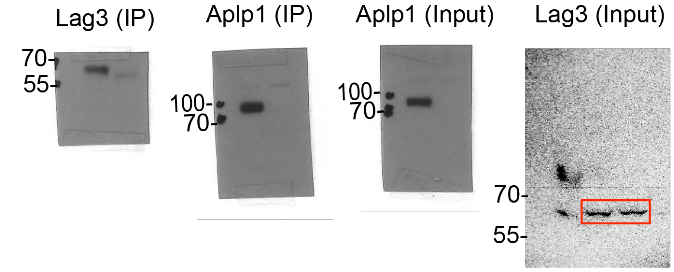

Figure 3c

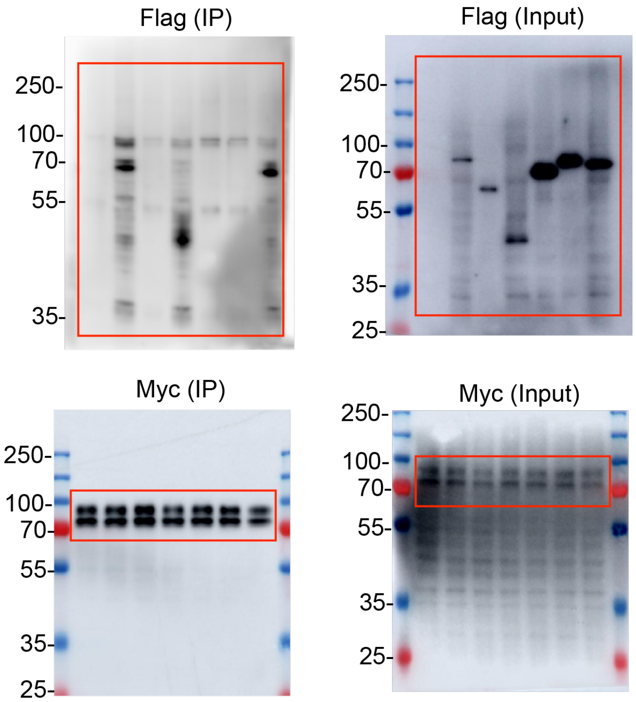

Figure 3d

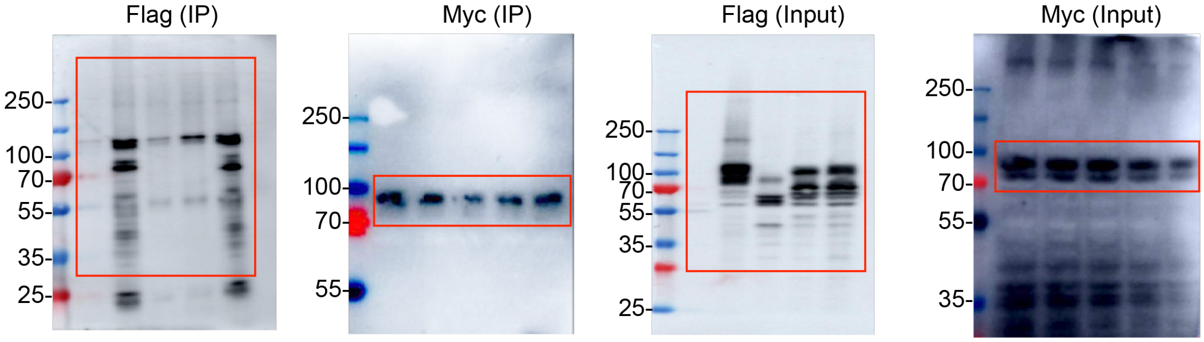

Figure 3e

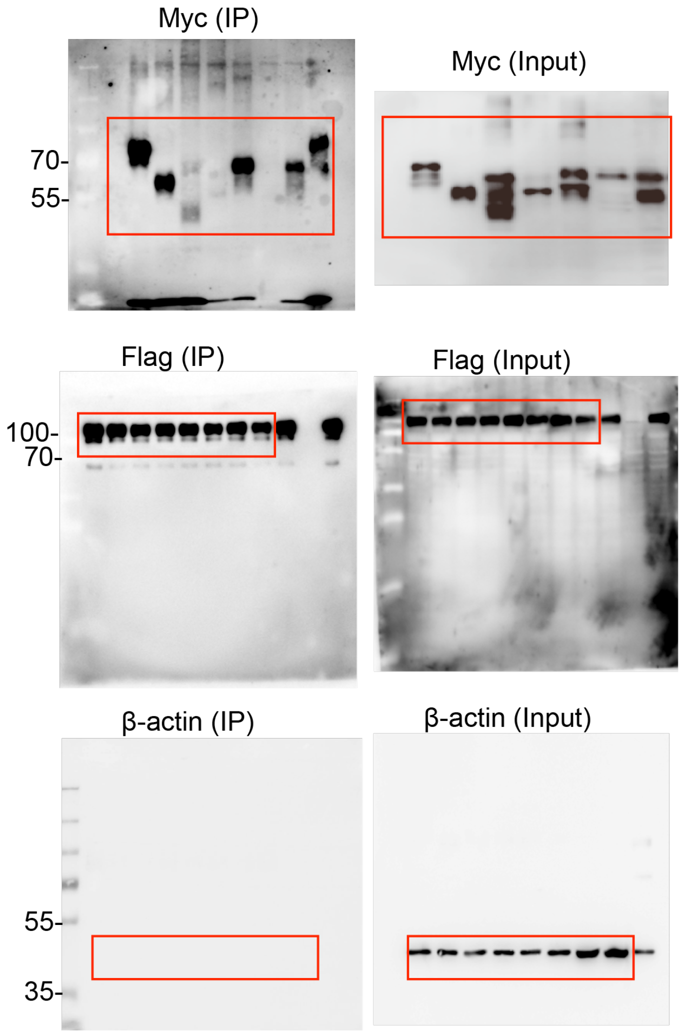

Figure 3i

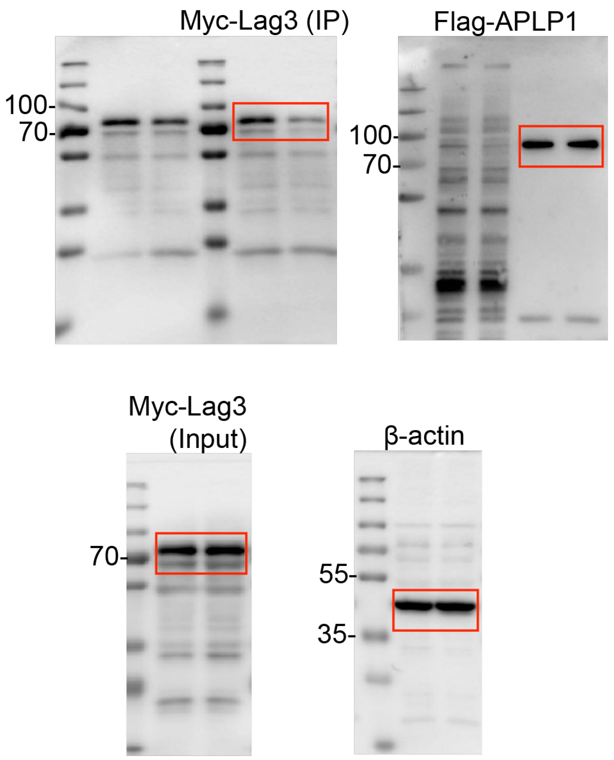

Figure 4c

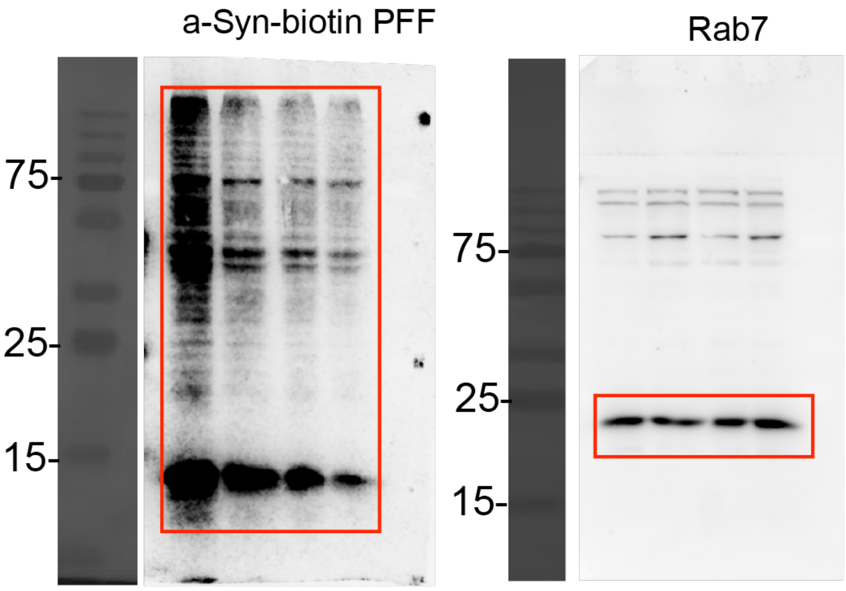

Figure 4h

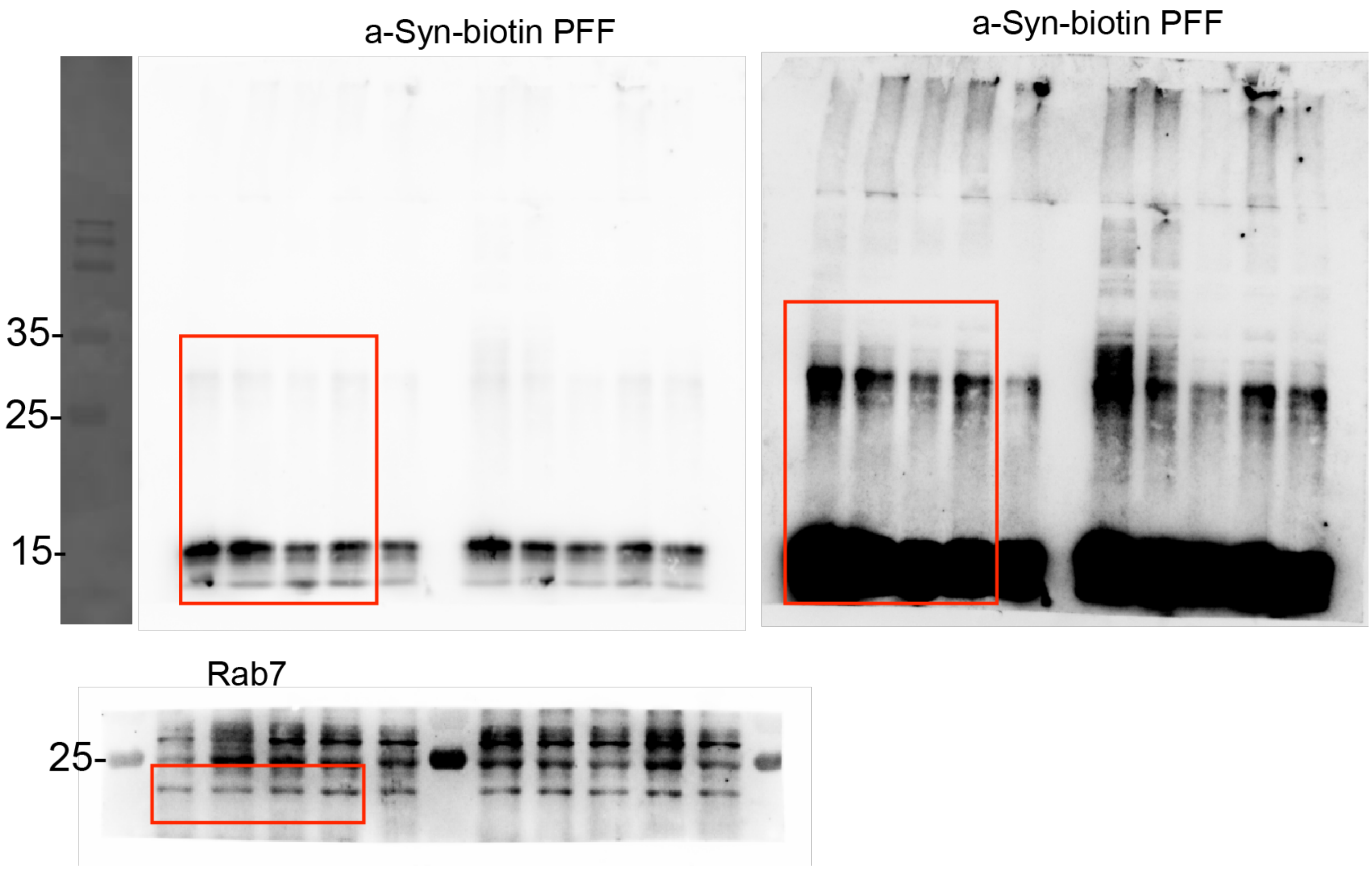

Figure 4f

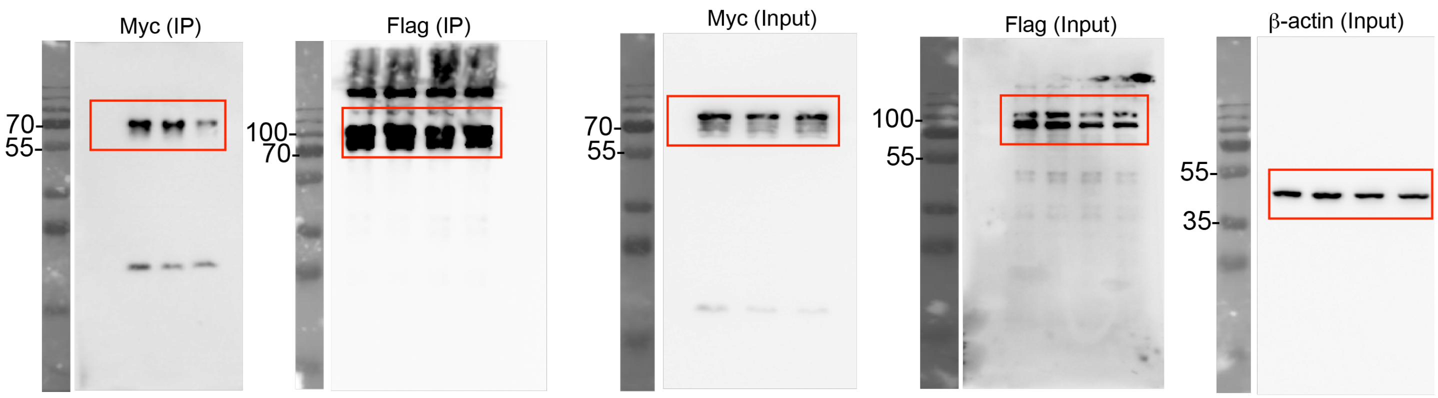

Figure 4j

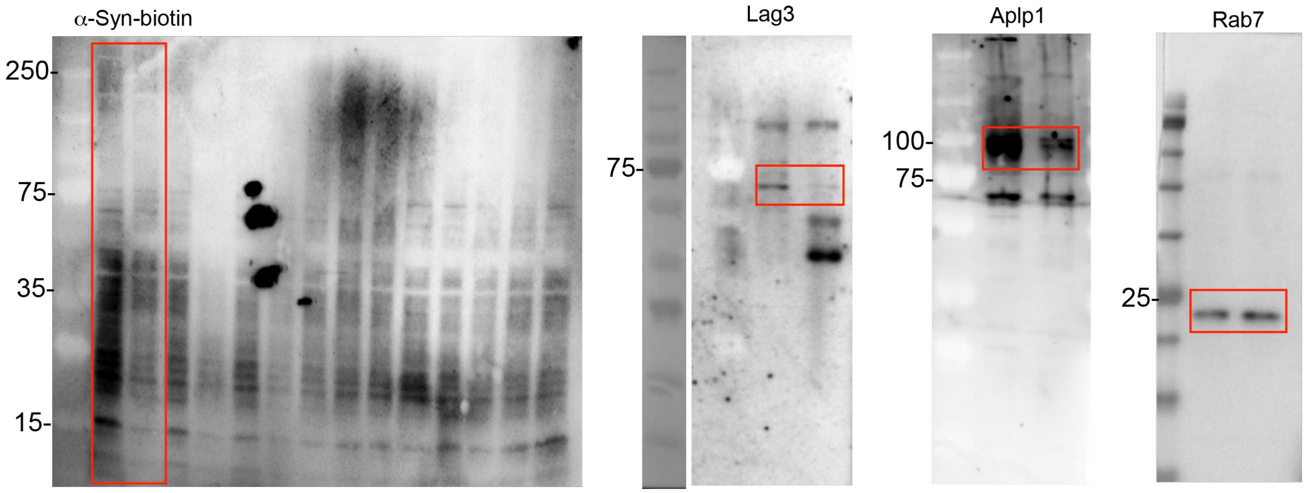

Figure S3b

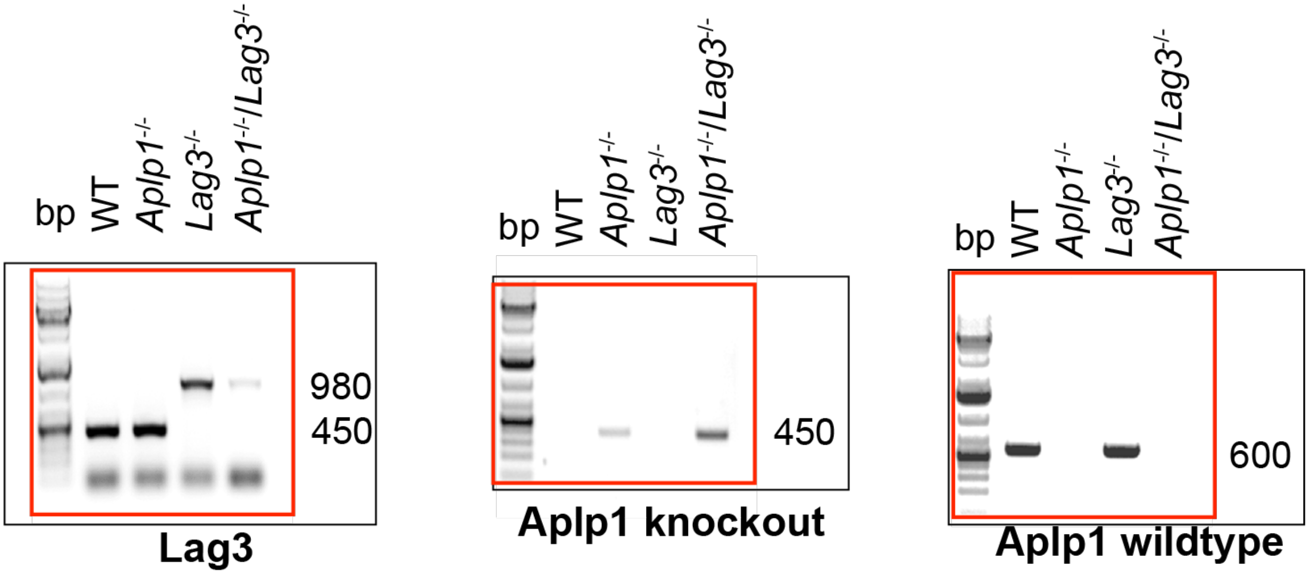
bp
WT
Aplp1-/-
Lag3-/-
Aplp1-/-/Lag3-/-
980
450
Lag3
bp
WT
Aplp1-/-
Lag3-/-
Aplp1-/-/Lag3-/-
450
Aplp1 knockout
bp
WT
Aplp1-/-
Lag3-/-
Aplp1-/-/Lag3-/-
600
Aplp1 wildtype

Figure S5e

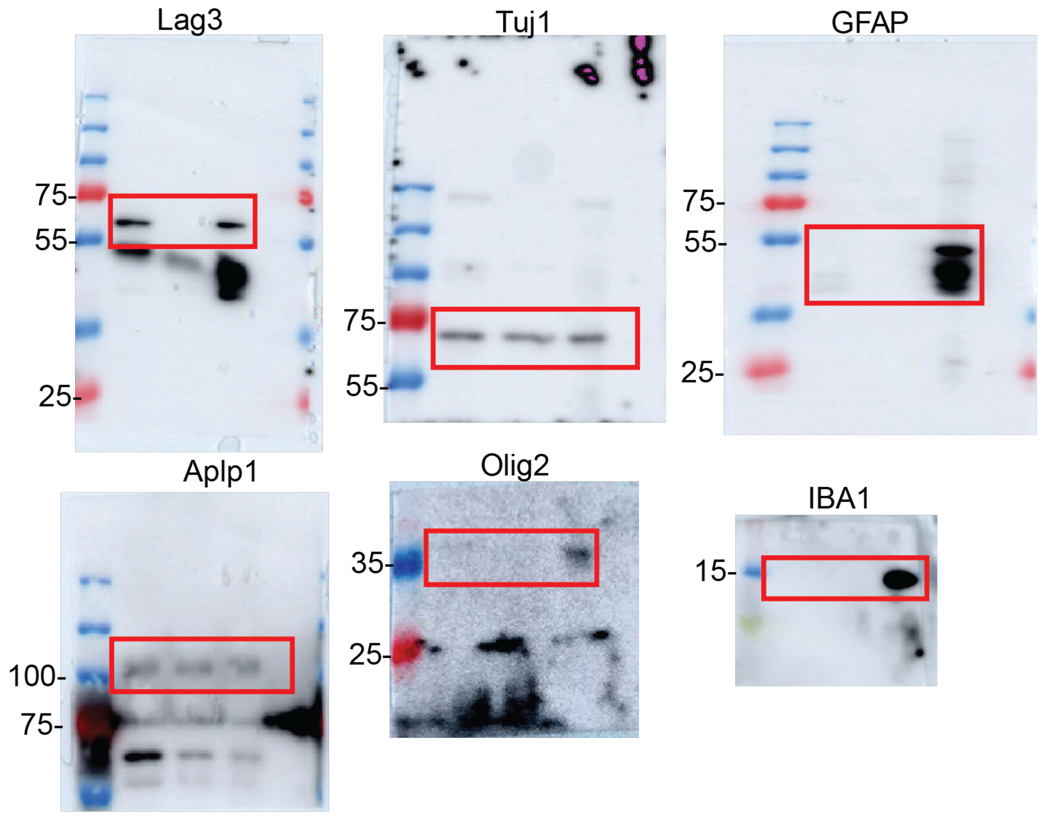

Figure S5f

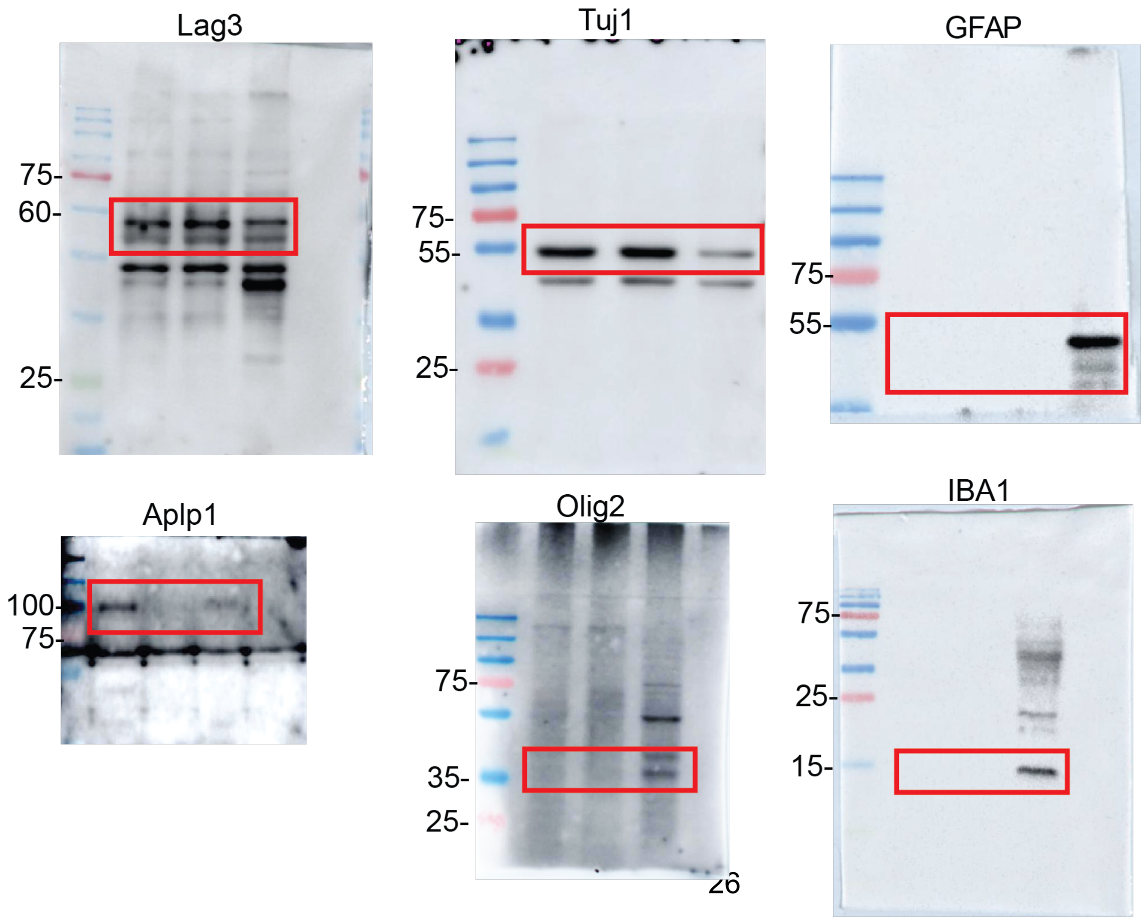

Figure S5d

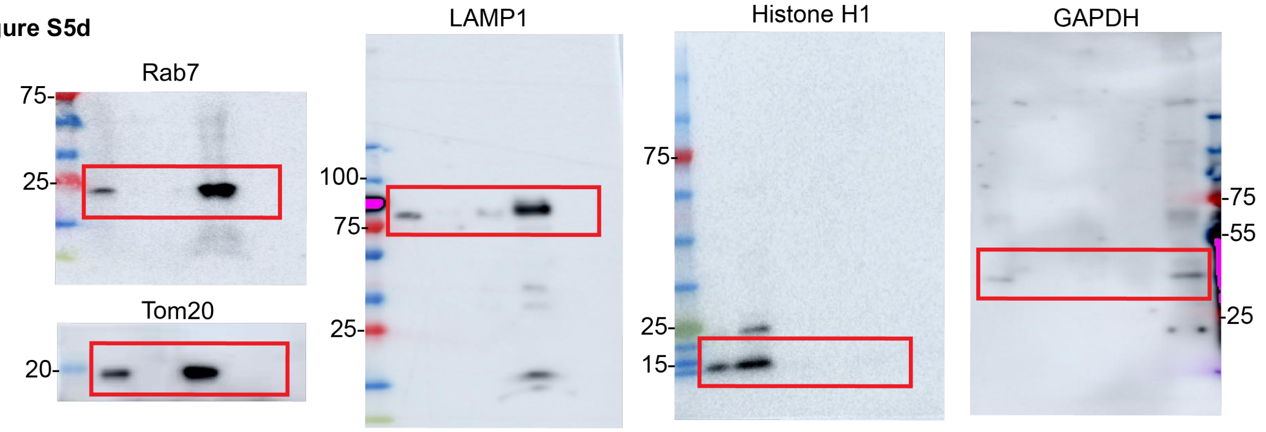

Figure S5g

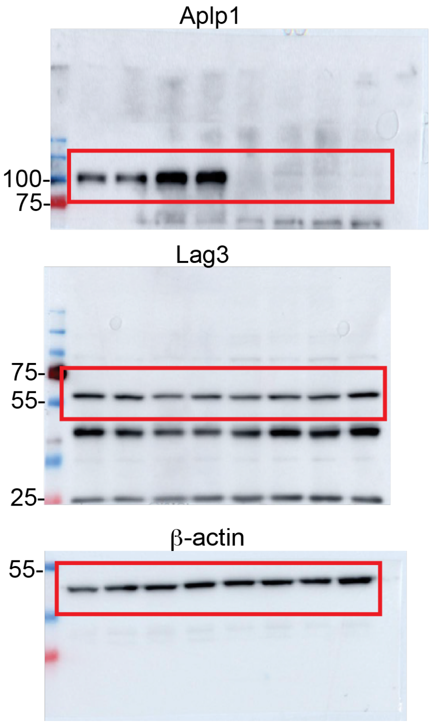

Figure S5h

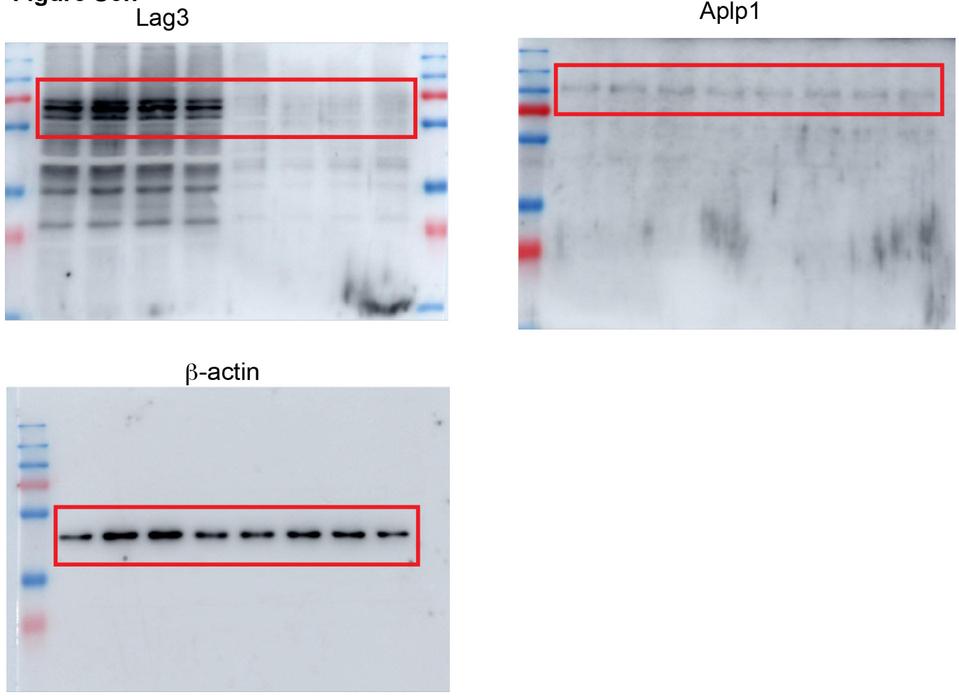

Figure S6d

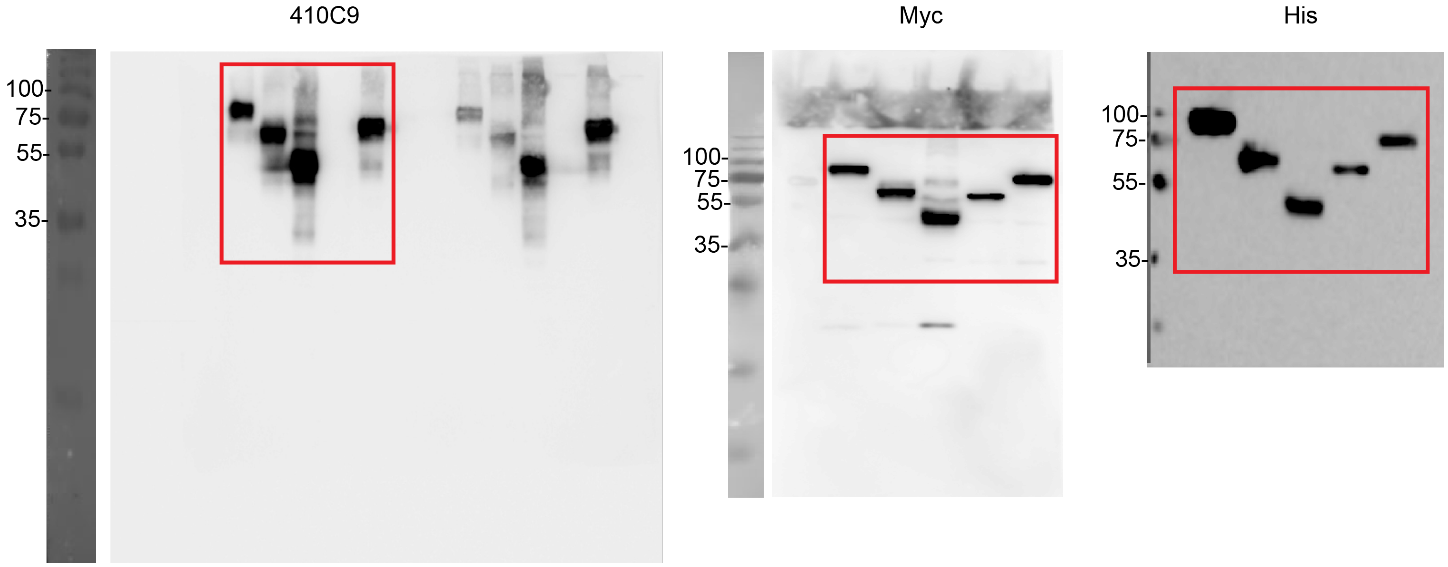

Figure S6e

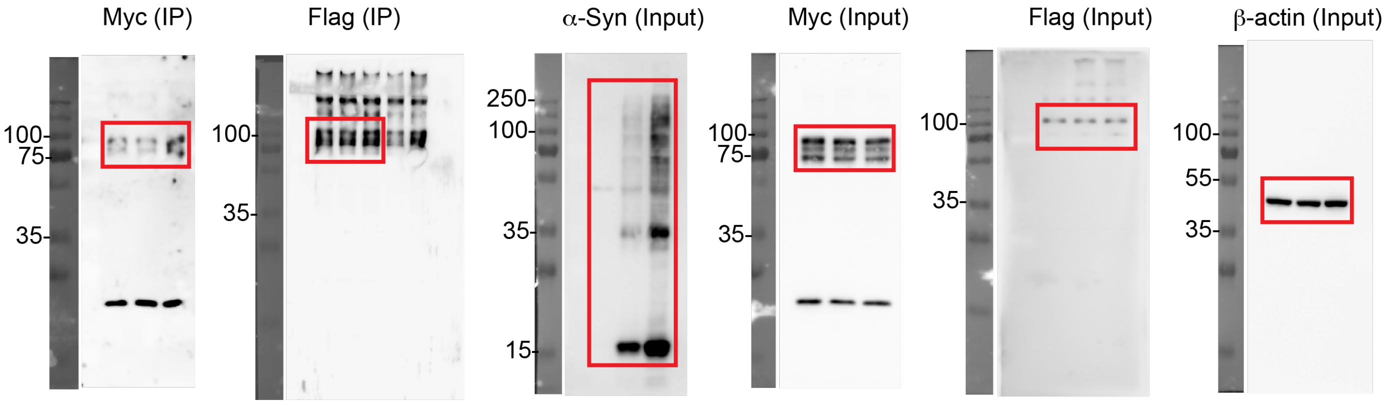

Figure S6f

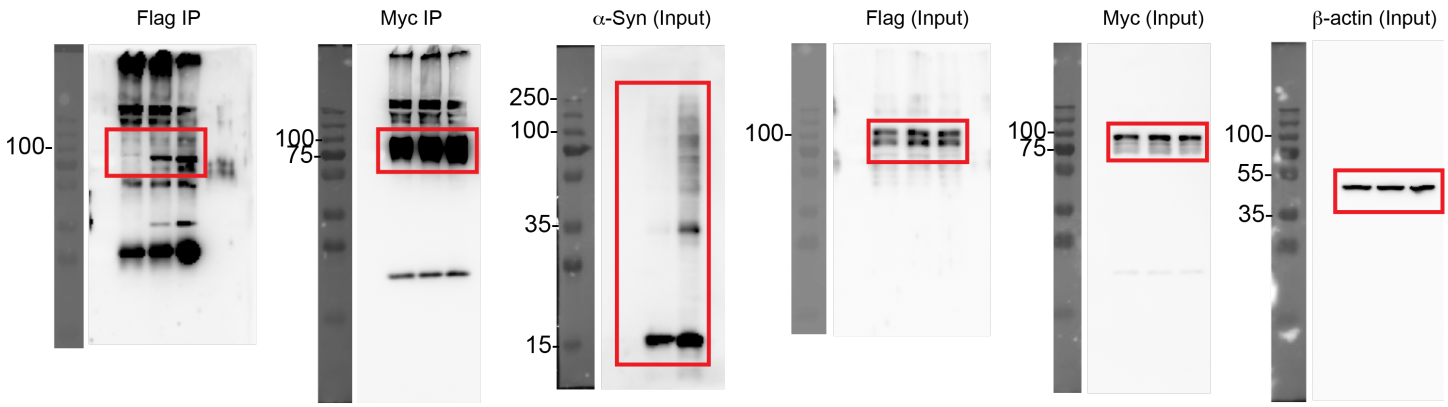

Figure S9k

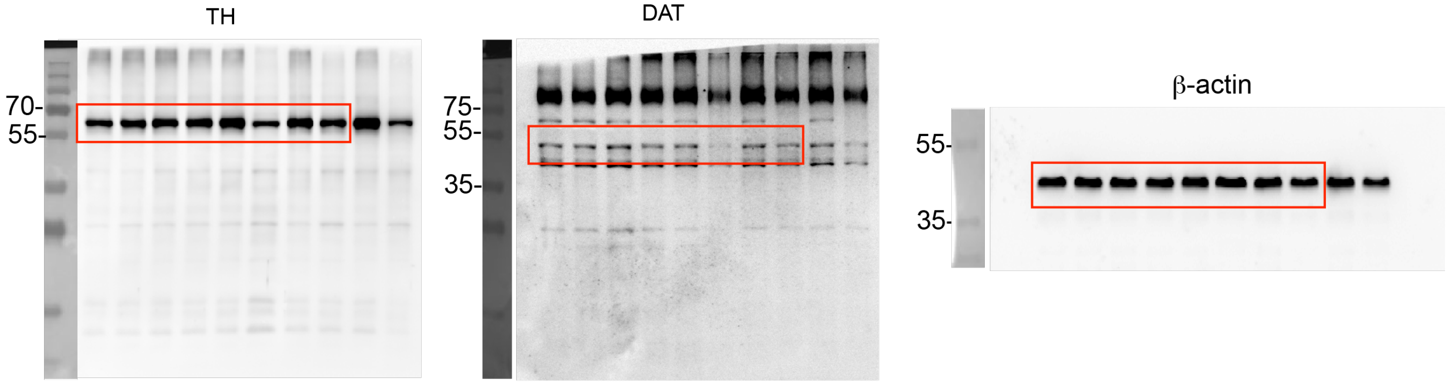

Supplement: Supplementary file 1 — Supplementary Information [file 41467_2024_49016_MOESM1_ESM.pdf]
