# Supplementary material for: Aplp1 interacts with Lag3 to facilitate transmission of pathologic α-synuclein
Source: Nat Commun. 2024 May 31;15:4663. doi: 10.1038/s41467-024-49016-3 (PMC11143359; doi:10.1038/s41467-024-49016-3)
Supplement: Supplementary file 3 — Reporting Summary [file 41467_2024_49016_MOESM3_ESM.pdf]

nature portfolio

Corresponding author(s): Xiaobo Mao, Han Seok Ko, Valina L. Dawson, Ted M. Dawson

Last updated by author(s): 01/05/2024

Reporting Summary

Nature Portfolio wishes to improve the reproducibility of the work that we publish. This form provides structure for consistency and transparency in reporting. For further information on Nature Portfolio policies, see our Editorial Policies and the Editorial Policy Checklist.

Statistics

For all statistical analyses, confirm that the following items are present in the figure legend, table legend, main text, or Methods section.

n/a	Confirmed	
☐	☒	The exact sample size (*n*) for each experimental group/condition, given as a discrete number and unit of measurement
☐	☒	A statement on whether measurements were taken from distinct samples or whether the same sample was measured repeatedly
☐	☒	The statistical test(s) used AND whether they are one- or two-sided<br>*Only common tests should be described solely by name; describe more complex techniques in the Methods section.*
☒	☐	A description of all covariates tested
☐	☒	A description of any assumptions or corrections, such as tests of normality and adjustment for multiple comparisons
☐	☒	A full description of the statistical parameters including central tendency (e.g. means) or other basic estimates (e.g. regression coefficient) AND variation (e.g. standard deviation) or associated estimates of uncertainty (e.g. confidence intervals)
☐	☒	For null hypothesis testing, the test statistic (e.g. *F*, *t*, *r*) with confidence intervals, effect sizes, degrees of freedom and *P* value noted<br>*Give P values as exact values whenever suitable.*
☒	☐	For Bayesian analysis, information on the choice of priors and Markov chain Monte Carlo settings
☒	☐	For hierarchical and complex designs, identification of the appropriate level for tests and full reporting of outcomes
☐	☒	Estimates of effect sizes (e.g. Cohen's *d*, Pearson's *r*), indicating how they were calculated

Our web collection on statistics for biologists contains articles on many of the points above.

Software and code

Policy information about availability of computer code

Data collection	Microscope Axio Observer Z1 , Zeiss Axiovision 4.6, Gen5 v3.11, Phillips CM 120 TEM,NMRPipe, ImageQuant LAS 4000mini scanner (GE Healthcare Life Sciences),Amersham Image 6000 (GE Healthcare Life Sciences)
Data analysis	ImageJ/FjJi software version 1.52p; Photoshop CS5 version 12.0.1 (Adobe); Illustrator version 23.0.6 (Adobe); GraphPad Prism software version 8.1.1, GraphPad Prism software<br>version 9,NMRView version 8.0.3, Zen lite 2012 (Blue edition), Amersham Image 6000, Microsoft Excel

For manuscripts utilizing custom algorithms or software that are central to the research but not yet described in published literature, software must be made available to editors and reviewers. We strongly encourage code deposition in a community repository (e.g. GitHub). See the Nature Portfolio guidelines for submitting code & software for further information.

Data

Policy information about availability of data

All manuscripts must include a data availability statement. This statement should provide the following information, where applicable:

- Accession codes, unique identifiers, or web links for publicly available datasets
- A description of any restrictions on data availability
- For clinical datasets or third party data, please ensure that the statement adheres to our policy

Data and materials availability: Further information and requests for resources and reagents should be directed to and will be fulfilled by the Lead Contact, Ted M.

Dawson (tdawson@jhmi.edu). There are no restrictions on any data or materials presented in this paper. All data are available in the main text or the Supplementary file. Source data are provided with this paper. Source files are available at: Dryad: https://doi.org/10.5061/dryad.5hqbzkhcw

Research involving human participants, their data, or biological material

Policy information about studies with human participants or human data. See also policy information about sex, gender (identity/presentation), and sexual orientation and race, ethnicity and racism.

Reporting on sex and gender	N/A
Reporting on race, ethnicity, or other socially relevant groupings	N/A
Population characteristics	N/A
Recruitment	N/A
Ethics oversight	N/A

Note that full information on the approval of the study protocol must also be provided in the manuscript.

Field-specific reporting

Please select the one below that is the best fit for your research. If you are not sure, read the appropriate sections before making your selection.

- [x] Life sciences
- [ ] Behavioural & social sciences
- [ ] Ecological, evolutionary & environmental sciences

For a reference copy of the document with all sections, see nature.com/documents/nr-reporting-summary-flat.pdf

Life sciences study design

All studies must disclose on these points even when the disclosure is negative.

Sample size	Sample size for cells and animal experiment was determined to be adequate based on experience of previous studies (PMID:27708076,30385548,35545089) and on literature describing similar experiments and consistency of measurable difference between the groups. All number of mice analyzed are reported in the figure Legends or supplementary methods of respective sections.<br>For in vitro experiments all experiments/measurements were performed in triplicate.
Data exclusions	Samples/animals were excluded from analysis in the instance of technical failure.
Replication	All the experiments were independently repeated and n number is provided in respective figure legends. All of the replicates showed similar results.
Randomization	Cells and animals were randomly assigned to different experimental groups. The animals used in the experiments were littermates. Mice were grouped according to genotype and randomly allocated for different experiments.
Blinding	Investigators were blinded during allocation, the conduct of the experiment, the outcome assessment, and the data analysis.

Reporting for specific materials, systems and methods

We require information from authors about some types of materials, experimental systems and methods used in many studies. Here, indicate whether each material, system or method listed is relevant to your study. If you are not sure if a list item applies to your research, read the appropriate section before selecting a response.

Materials & experimental systems

n/a	Involved in the study	
[ ]	[x]	Antibodies
[ ]	[x]	Eukaryotic cell lines
[x]	[ ]	Palaeontology and archaeology
[ ]	[x]	Animals and other organisms
[x]	[ ]	Clinical data
[x]	[ ]	Dual use research of concern
[x]	[ ]	Plants

Methods

n/a	Involved in the study	
[x]	[ ]	ChIP-seq
[x]	[ ]	Flow cytometry
[x]	[ ]	MRI-based neuroimaging

Antibodies

Antibodies used	We provided the list of all antibodies including the supplier and catalog number in the supplementary table 2 (Supplementary data) and listed below:<br>Mouse anti-lymphocyte-activation gene 3 (Lag3) (Dario Vignali lab and Millipore Sigma, 410C9 (4-10-C9), MABF954)<br>Rabbit anti-amyloid β precursor like protein 1 (Aplp1) (Gopal Thinakaran lab, A1NT)<br>Rabbit anti-amyloid β precursor like protein 1 (Aplp1) (Gopal Thinakaran lab, CT11)<br>Mouse anti-α-synuclein (BD Bioscience, Cat# 610787, RRID:AB_398108)<br>Rabbit anti-pS129-α-synuclein (Abcam, Cat# ab51253, RRID:AB_869973)<br>Mouse anti-neuronal nuclei (NeuN) (Millipore Sigma, Cat# MAB377, RRID:AB_2298772)<br>Rabbit anti-Tyrosine Hydroxylase (TH) (Novus Biologicals, Cat# NB300-109, RRID:AB_10077691)<br>Mouse anti-Myc-HRP (Cell Signaling Technology, Cat# 2040S, RRID:AB_2148465)<br>Rabbit anti-Rab5 (Abcam, Cat# ab18211, RRID:AB_470264)<br>Rabbit anti-GAPDH-HRP (Cell Signaling Technology Cat# 3683S, RRID: AB_1642205)<br>Rabbit anti-Rab7 (Cell Signaling Technology, Cat# 2094, RRID:AB_2300652)<br>Rabbit anti-lysosomal associated membrane protein 1 (LAMP1) (Abcam, Cat# ab24170, RRID:AB_775978)<br>Rabbit anti-Clathrin (Abcam, Cat# ab59710, RRID:AB_941047)<br>Rabbit anti-Dynamin II (Abcam, Cat# ab3457, RRID:AB_2093679)<br>Rabbit anti-Caveolin-1 (CAV-1) (Abcam, Cat# ab2910, RRID:AB_303405)<br>Mouse anti-β-actin (ThermoFisher Scientific, Cat# MA5-15739-HRP, RRID: AB_2537667)<br>Mouse anti-microtubule-associated protein 2 (MAP2) (Sigma, Cat# M9942, RRID:AB_477256)<br>Rabbit anti-MAP2 (Millipore Sigma, Cat# AB5622, RRID:AB_91939)<br>Rabbit Tuj1 – (Cell signalling technology, β3-tubulin (D71G9) Cat# 5568, RRID: AB_10694505)<br>Flag-HRP - anti-FLAG (Cell Signaling Technology, Cat# 14793S)<br>Rabbit anti-ionized calcium binding adaptor molecule 1 (IBA1) (Wako, Cat# 019-19741, RRID: AB_839504)<br>Rabbit anti-glial fibrillary acidic protein (GFAP) (Abcam, Cat# ab7260, RRID:AB_305808)<br>Mouse anti-GFP (ThermoFisher Scientific, Cat# MA5-15256, RRID: AB_10979281)<br>Donkey Anti-Rabbit IgG, Whole Ab ECL Antibody, HRP Conjugated (GE Healthcare, Cat#NA934; RRID: AB_772206)<br>Sheep Anti-Mouse IgG, Whole Ab ECL Antibody, HRP Conjugated (GE Healthcare, Cat#NA931; RRID: AB_772210)
Validation	Antibodies used in this study are validated by manufacturer,PIRDs and in the cited articles. Validation data for commercial antibodies are available on vendor websites. Appropriate controls were used in every experiment whenever possible to ensure validity of used antibodies in this study.

Eukaryotic cell lines

Policy information about cell lines and Sex and Gender in Research

Cell line source(s)	Human HEK293FT cells (Invitrogen, Cat#: R70007; RRID:CVCL_6911)<br>SH-SY5Y cells (ATCC, cat#: CRL-2266)<br>410C9 hybridoma cell line was obtained from Dr. Dario Vignali from University of Pittsburgh.
Authentication	HEK293FT and SH-SY5Y cell lines were authenticated based on morphology, cell replicate rate and cell transfection efficiency every 3 months. No misidentified cell line was used in this study. Cell lines were not authenticated by other means than listed here.
Mycoplasma contamination	The cell lines were routinely checked for Mycoplasma. All cell lines tested negative for mycoplasma contamination.
Commonly misidentified lines<br>(See ICLAC register)	No commonly misidentified cell lines were used in this study.

Animals and other research organisms

Policy information about studies involving animals; ARRIVE guidelines recommended for reporting animal research, and Sex and Gender in Research

Laboratory animals	C57BL/6 WT were obtained from the Jackson Laboratories (Bar Harbor, ME).<br>Lag3–/– mice with C57BL/6 background were obtained from Dr. Charles G. Drake when he was at Johns Hopkins University.<br>Aplp1–/– mice with C57BL/6 background were obtained from Dr. Ulrike Muller at the University of Heidelberg.<br>Aplp1–/–/Lag3–/– double knockout mice were generated by two consecutive crosses. Aplp1–/– and Lag3–/– were intercrossed to obtain the Aplp1+/–/Lag3+/– mice. These animals were further intercrossed (Aplp1+/–/Lag3+/– × Aplp1+/–/Lag3+/–) to obtain double knockout in the next generation.<br>Lag3 L/L-YFP mice were obtained from Dr. Dario Vignali from University of Pittsburgh.<br><br>All mice were housed under standard conditions of constant temperature of (22± 1 degree Celcius), relative humidity of 42% and 12 hour light cycle with food and water.

Wild animals	No wild animals were used in this study.
Reporting on sex	Both sexes were used in the experiments; No sex based analysis was performed in this study. Number of animals used in each experiments are listed in respective figure legends.
Field-collected samples	No field-collected samples were used in this study.
Ethics oversight	All procedures were carried out with approval from the the Johns Hopkins University Animal Care and Use Committee (ACUC) and in accordance with the National Institutes of Health (NIH) Guidelines for the Care and Use of Experimental Animals.

Note that full information on the approval of the study protocol must also be provided in the manuscript.

Plants

Seed stocks	N/A
Novel plant genotypes	N/A
Authentication	N/A
